# Quantitative Analysis of Protein Evolution: The Phylogeny of Osteopontin

**DOI:** 10.3389/fgene.2021.700789

**Published:** 2021-08-16

**Authors:** Xia Wang, Georg F. Weber

**Affiliations:** ^1^Department of Mathematical Sciences, University of Cincinnati, Cincinnati, OH, United States; ^2^Academic Health Center, University of Cincinnati, Cincinnati, OH, United States

**Keywords:** phylogeny, phylogenetic tree, protein sequence, complex systems, autocorrelation, average mutual information, fractal dimension, wavelet analysis

## Abstract

The phylogenetic analysis of proteins conventionally relies on the evaluation of amino acid sequences or coding sequences. Individual amino acids have measurable features that allow the translation from strings of letters (amino acids or bases) into strings of numbers (physico-chemical properties). When the letters are converted to measurable properties, such numerical strings can be evaluated quantitatively with various tools of complex systems research. We build on our prior phylogenetic analysis of the cytokine Osteopontin to validate the quantitative approach toward the study of protein evolution. Phylogenetic trees constructed from the number strings differentiate among all sequences. In pairwise comparisons, autocorrelation, average mutual information and box counting dimension yield one number each for the overall relatedness between sequences. We also find that bivariate wavelet analysis distinguishes hypermutable regions from conserved regions of the protein. The investigation of protein evolution via quantitative study of the physico-chemical characteristics pertaining to the amino acid building blocks broadens the spectrum of applicable research tools, accounts for mutation as well as selection, gives assess to multiple vistas depending on the property evaluated, discriminates more accurately among sequences, and renders the analysis more quantitative than utilizing strings of letters as starting points.

## Introduction

The analysis of amino acid sequences or coding sequences for proteins is central to the study of molecular evolution. Among the main tools is the construction of phylogenetic trees, which are assembled based on algorithms that consider the numbers of mismatched amino acids or bases between aligned sequences (Charleston, 2013; He, 2019). In this strategy, multiple comparisons may be characterized by identical numbers of differences and are placed on the same evolutionary level. The resultant tree represents a hypothesis on the developmental paths that have led to the existing diversity.

The rationale for basing evolutionary studies of proteins on their differences in amino acid sequences is rooted in the mechanism of mutation, which alters bases in the coding sequence, consecutively resulting in alterations of individual amino acids. However, selection–the second major driver of evolution–favors certain mutations over others, and this preference is not captured by the evaluation of sequence mismatches. It is feasible to research protein evolution by taking the selection component into account, which may be best reflected in the physico-chemical properties of the mutated and selected amino acids. We hypothesized that basing phylogenetic analysis on such quantifiable properties of amino acids is more discriminating than predicating this analysis on base or amino acid sequences. By incorporating a readout for selection, not only mutation, evolutionary distances calculated through this quantitative method are likely more accurate than distance estimates obtained from conventional approaches.

The replacement of the letters, which indicate identities or mismatches of the amino acids, with quantitative measurements of their properties generates strings of numbers that can be studied with various mathematical techniques of complex systems analysis. Therefrom, numerical assessments are obtainable for the pairwise relatedness of these strings of numbers, including autocorrelation, average mutual information, and fractal dimension. More quantitative phylogenetic trees can thus be generated. Bivariate wavelet analysis differentiates hypermutable from conserved regions. Here we apply these techniques to the evaluation of Osteopontin phylogeny.

The cytokine Osteopontin is of keen interest because of its importance for tissue remodeling, cellular immune responses, and calcium homeostasis in milk and urine. In pathophysiology, the biomolecule contributes to the progression of multiple cancers (Weber, 2008). From the evolutionary context, conserved and variable domains can be inferred. It is implied that domains with a high level of conservation among species are reflective of important biological functions being fulfilled by such regions. A comprehensive phylogenetic analysis of 202 Osteopontin protein sequences was recently conducted with conventional methods (Weber, 2018). It followed a much more basic tree construction reported earlier (Sivakumar and Devaraj, 2014). Here, we go beyond those predecessor studies with a quantitative evaluation based on the physico-chemical properties of the Osteopontin amino acid strings.

## Methods

### Source Data

To study Osteopontin, we have used a subset of taxa from Weber (2018), having eliminated the sequences belonging to fish, birds, reptiles and rodents. As we develop new approaches for phylogenetic analysis in the present investigation, we sought to focus the large number of available sequences only on comparisons among higher-developed taxa. The less extensive range of diversity in this domain also allows us to concentrate on more nuanced sequence variations. We constructed consensus sequences for each taxonomic group under study here (primata, perissodactyla, carnivora, chiroptera, artiodactyla a = camelidae/suidae/celaceae, artiodactyla b = cervidae/bovidae, marsupialia, prototheria, xenarthra/afroteria) by applying the most common amino acid to all polymorphic sites, then aligning them in Clustal Omega.

To corroborate the methodology, we performed a separate analysis on the avian Osteopontin sequences. Five major clades of neoaves have been described (Prum et al., 2015). Among 64 avian Osteopontin sequences in NCBI nucleotide, we previously identified four groups. We constructed consensus sequences for each taxonomic group by choosing the most common amino acid for every polymorphic site, aligned the sequences with Clustal Omega, converted them into number strings according to their physico-chemical properties (gaps were replaced with 0 yielding number strings of 332 positions for all groups), and calculated the distances on phylogenetic trees. We also constructed a tree based on the autocorrelation values.

To confirm that the approach is generally applicable, we further tested another protein. The phylogeny of Vascular Endothelial Growth Factor (VEGF) has been studied with conventional methods (Holmes and Zachary, 2005; He et al., 2014). VEGF coding sequences were retrieved from Homo sapiens (GenBank: AY047581.1), Piliocolobus tephrosceles (NCBI: XP_023068672.1), Sus scrofa (GenBank: JF831364.1), Spalax ehrenbergi (GenBank: AF186236.1), Xenopus laevis (GenBank: AF008594.1), Passer montanus (GenBank: KY001973.1), Gallus gallus (GenBank: AB011078.1), Trimeresurus flavoviridis (GenBank: AB154419.1), Bovine papular stomatitis virus strain V660 (GenBank: AY513237.1), Parapoxvirus of red deer strain RD86 (GenBank: DQ888328.1). We applied autocorrelation, average mutual information, and harmonic analysis.

### Sequence Conversion

The values for select properties of the amino acids were used to replace the letter codes, so as to characterize volume, hydropathy index, solubility, octanol interface, or pI at 25°C. Even though the exact numbers pertaining to the properties of the isolated amino acids may shift after their incorporation into a protein, they represent good estimates for quantification of the molecular characteristics. Gaps were replaced by 0, yielding number strings of 356 entries for the Osteopontins from the advanced taxa.

### Phylogenetic Tree

Because available programs to generate phylogenetic trees require the input of letter strings, a manual approach had to be applied for this study. We generated phylogenetic trees with a strategy similar to conventional algorithms (Meneely et al., 2017). For each physico-chemical property under study, the number strings pertaining to the Osteopontins were aligned pairwise, such that all pairs were covered. In each position (1 through 356 for advanced species, 1 through 332 for aves, 1 through 230 for VEGF), the absolute of the difference between two sequences was calculated, and the results for all positions were added up to yield the sum-difference. Among all the pairwise comparisons, the smallest sum-difference between two strings of numbers was considered to represent the evolutionary distance between the closest relatives. In the next step, the number strings for the two taxa with the smallest sum-difference were combined by averaging at each position, and the process was repeated until all distances between taxa had been calculated. The tree was constructed with the branch lengths equaling the calculated distances.

### Autocorrelation

Proteins at various stages of evolution sometimes repeat patterns or have other properties, according to which developmentally earlier values display some relation to later values. The autocorrelation statistic measures the degree of that affiliation as it refers to linear dependence. The magnitude of its dimensionless number reflects the extent of similarity. The formula for autocorrelation *R*_m_ is comprised of terms for autocovariance and variance

$$autocorelation=\frac{autocovariance}{variance}$$

$$R_{m}=\frac{\frac{1}{N}\sum_{\text{t}=1}^{N-m}\left(x_{t}-\bar{x}\right)\left(x_{t+m}-\bar{x}\right)}{\frac{1}{N}\sum_{\text{t}=1}^{\text{N}}{\left(x_{t}-\bar{x}\right)}^{2}}$$

Autocorrelation coefficients range from −1 to +1, with +1 indicating perfect synchrony and −1 reflecting exact mirror images. An absence of any correlation yields *R*_m_ = 0.

### Average Mutual Information

The average mutual information, an information theory measure, summarizes a non-linear correlation function that quantifies the amount of information shared between the sequence data from two species. The average mutual information was calculated with the mi.empirical function in the R package entropy, where the Shannon entropy is calculated. The mutual information, MI, is defined as MI = H(X)+H(Y)–H(X,Y), where H(.) represents the marginal or the joint entropy function.

### Box Counting Dimension

Contrasting with Euclidean measures, the dimension in terms of fractal geometry is best described as a non-integer. This dimension serves as a quantitative measure for the evaluation of geometric complexity by objects, such as two number strings representing different taxa. A general relationship assumes

$$dimension\propto \frac{\log\left(\mathrm{number}\mathrm{of}\mathrm{increments}\right)}{\log\left(\frac{1}{\mathrm{scale}\mathrm{size}}\right)}$$

Here, we estimated the box counting dimension for the plots of pairwise comparisons between taxa after binning into 10 × 10 squares of 2-dimensional graphs (with both axes of identical length). The numbers generated as indicators of dimension are larger than 1 (approximating identity between the taxa) and smaller than 2 (approximating total independence between the sequences). Smaller box counting dimensions are reflective of closer relatedness between the number strings representing two taxa.

### Bivariate Wavelet Analysis

The cross-wavelet analysis allows us to conduct pairwise comparison between taxa from the frequency domain. It illustrates the similarity in periodicity between two sequences. We have four images to summarize the analysis results, including the cross-wavelet power plot, the wavelet coherence plot, the average power plot and the phase difference image. While cross-wavelet power corresponds to covariance, wavelet coherence is a measure similar to correlation with a value range between 0 and 1. Two sequences are coherent in periodicity if they have a constant relative phase. The cross-wavelet power and coherence plots also contain arrows showing the area of significant joint periods (significance level = 0.05). The direction of the arrows indicates the direction of phase differences with (π/2, π) and (−π, −π/2) angles for out-of-phase and (−π/2, π/2) for in-phase. A more explicit global view of the phase difference is shown in the phase difference image. The coherence plot, which is not affected by wide swings in sequences, is more appropriate in showing the areas where two sequences share jointly significant periods. The cross-wavelet average power plot shows the shared periods, the corresponding average power and the corresponding statistical significance.

## Results

### Physico-Chemical Properties of Amino Acids

There are empirical numbers for all twenty amino acids, which are used in higher species, to characterize their molecular weights, sizes, volumes, various solubility characteristics, and isoelectric points (Table 1). These numbers represent good estimates for quantification of the relevant properties. For each taxon under investigation, the consensus Osteopontin sequence was generated by choosing the most common amino acid for every polymorphic site, and the sequences were aligned with Clustal Omega (Supplementary Table 1). Gaps were replaced by 0 (yielding number strings of 356 entries for all taxa), and all amino acids were replaced by the number for the physico-chemical characteristic of interest (volume, hydropathy index, solubility, octanol interface, and pI at 25°C) (for solubility as an example, see Supplementary Figure 1).

**TABLE 1:** Physico-chemical properties of individual amino acids.

			**Da**	**Angstrom**	**Angstrom**			**g/kgH_2_O**		**ΔGwoct (kcal/mol)**	**ΔGwif (kcal/mol)**			
**Amino acid**	**Code**	**One letter**	**Molecular mass**	**Surface**	**Volume**	**Hydropathy index**	**Hydrophobicity**	**Solubility**	**Octanol–interface scale**	**Octanol scale**	**Interface scale**	**pI at 25°C**	**pKa, NH2**	**pKa, COOH**
Alanine	Ala	A	89.094	115	88.6	1.8	0.616	166.9	0.33	0.5	0.17	6.11	9.87	2.35
Arginine	Arg+	R	174.203	225	173.4	−4.5	0	182.6	1	1.81	0.81	10.76	9.09	2.18
Asparagine	Asn	N	132.119	150	111.1	−3.5	0.236	25.1	0.43	0.85	0.42	10.76	8.8	2.02
Aspartate	Asp−	D	133.104	160	114.1	−3.5	0.028	5.04	2.41	3.64	1.23	2.98	9.6	1.88
Cysteine	Cys	C	121.154	135	108.5	2.5	0.68	277	0.22	−0.02	−0.24	5.02	10.78	1.71
Glutamate	Gln	E	147.131	190	138.4	−3.5	0.043	8.6	0.19	0.77	0.58	3.08	9.67	2.19
Glutamine	Glu−	Q	146.146	180	143.8	−3.5	0.251	42	1.61	3.63	2.02	5.65	9.13	2.17
Glycine	Gly	G	75.067	75	60.1	−0.4	0.501	239	1.14	1.15	0.01	6.06	9.6	2.34
Histidine	His+	H	155.156	195	153.2	−3.2	0.165	43.5	1.37	2.33	0.96	7.64	8.97	1.78
Isoleucine	Ile	I	131.175	175	166.7	4.5	0.943	34.2	−0.81	−1.12	−0.31	6.04	9.76	2.32
Leucine	Leu	L	131.175	170	166.7	3.8	0.943	23.8	−0.69	−1.25	−0.56	6.04	9.6	2.36
Lysine	Lys+	K	146.189	200	168.6	−3.9	0.283	5.8	1.81	2.8	0.99	9.47	10.28	2.18
Methionine	Met	M	149.208	185	162.9	1.9	0.738	56	−0.44	−0.67	−0.23	5.74	9.21	2.28
Phenylalanine	Phe	F	165.192	210	189.9	2.8	1	27.9	−0.58	−1.71	−1.13	5.91	9.24	2.58
Proline	Pro	P	115.132	145	112.7	−1.6	0.711	50	−0.31	0.14	0.45	6.3	10.6	1.99
Serine	Ser	S	105.093	115	89	−0.8	0.359	250	0.33	0.46	0.13	5.68	9.15	2.21
Threonine	Thr	T	119.119	140	116.1	−0.7	0.45	90.6	0.11	0.25	0.14	5.6	9.12	2.15
Tryptophan	Trp	W	204.228	225	227.8	−0.9	0.878	13.2	−0.24	−2.09	−1.85	5.88	9.39	2.38
Tyrosine	Tyr	Y	181.191	230	193.6	−1.3	0.88	0.51	0.23	−0.71	−0.94	5.63	9.11	2.2
Valine	Val	V	117.148	155	140	4.2	0.825	88	−0.53	−0.46	0.07	6.02	9.72	2.29

*The table shows the amino acid name, code, and one-letter abbreviation, followed by the values for the indicated physico-chemical properties. The top row specifies applicable units.*

### Phylogenetic Trees

**FIGURE 1:**
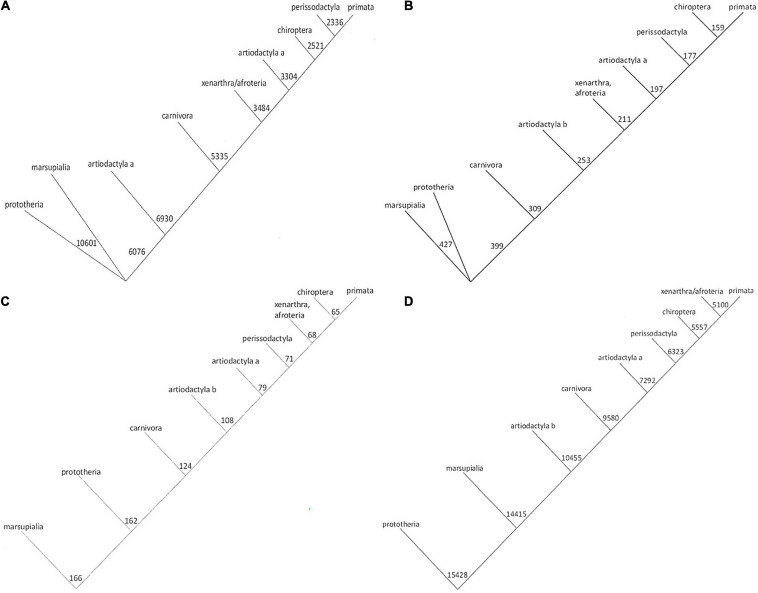
Phylogenetic trees for Osteopontin evolution. The amino acid sequences were converted to strings of numbers. Phylogenetic trees were generated on the basis of calculating the sum differences between all pairs of strings, such that at each step the two strings with the smallest sum-difference between them were averaged before moving to the next step and repeating the process. The numbers express the distances as calculated from the indicated properties, the lengths of the lines are drawn to scale (they are proportional to the numerical differences calculated). The trees in panels **(A,B)** versus panels **(C,D)** offer distinct hypotheses for evolutionary relatedness. **(A)** Tree based on amino acid volume. **(B)** Tree based on amino acid hydropathy. **(C)** Tree for octanol interface. **(D)** Tree for solubility.

There are several algorithms in use for the construction of conventional phylogenetic trees, yielding similar but distinct results (see examples in Supplementary Figure 2). Applying a more quantitative strategy, for each physico-chemical property under study, we utilized the number strings to calculate relatedness, and assemble numerical trees. The trees based on amino acid volume and hydropathy are very similar to each other (Figures 1A,B). Likewise, the trees based on solubility and octanol interface resemble each other closely (Figures 1C,D). These two groups are reflective of alternative hypotheses regarding the evolution of Osteopontin. Noticeably, the trees generated in this study (Figure 1 and Supplementary Figure 5–described below) are mutually less divergent than the trees generated with the conventional algorithms (Supplementary Figure 2).

We corroborated the approach with the separate subset of avian Osteopontin sequences (Supplementary Figure 6, deliberately placed at the end). Strikingly, the conventional trees displayed stark divergence, whereas the trees generated on the basis of physico-chemical properties or on the basis of their autocorrelation (averaged across all properties) were highly consistent.

### Overall Relatedness

Readouts of complexity allow for pairwise comparisons between two strings of numbers. The protein sequences under study here were analyzed according to their autocorrelation, average mutual information, and box counting dimension (an estimate of their fractal dimension).

The autocorrelation of two data strings with few mutations is expected to be high (approaching 1.0). Thus, increasing evolutionary distance can be reflected in a reduction of the autocorrelation. We calculated the autocorrelation values, pairwise between taxa, for each physico-chemical parameter under study (Supplementary Table 2A) and averaged all values as estimates for evolutionary relatedness (Table 2A).

**TABLE 2:** Overall relatedness of Osteopontin sequences among taxa.

**A**		**Primata**	**Perissodactyla**	**Xenarthra/afroteria**	**Chiroptera**	**Artiodactyla a**	**Artiodactyla b**	**Carnivora**	**Marsupialia**	**Prototheria**
	Primata		0.848 ± 0.058	0.831 ± 0.039	0.857 ± 0.024	0.813 ± 0.045	0.716 ± 0.042	0.803 ± 0.077	0.521 ± 0.060	0.499 ± 0.057
	Perissodactyla			0.836 ± 0.038	0.851 ± 0.040	0.832 ± 0.046	0.690 ± 0.107	0.759 ± 0.028	0.557 ± 0.069	0.503 ± 0.067
	Xenarthra/afroteria				0.835 ± 0.033	0.807 ± 0.044	0.710 ± 0.090	0.719 ± 0.018	0.541 ± 0.057	0.564 ± 0.125
	Chiroptera					0.814 ± 0.031	0.718 ± 0.146	0.744 ± 0.037	0.508 ± 0.079	0.490 ± 0.076
	Artiodactyla a						0.738 ± 0.048	0.750 ± 0081	0.559 ± 0.078	0.548 ± 0.064
	Artiodactyla b							0.610 ± 0.081	0.447 ± 0.141	0.485 ± 0.137
	Carnivora								0.520 ± 0.051	0.506 ± 0.027
	Marsupialia									0.548 ± 0.050
	Prototheria									
**B**		**Primata**	**Perissodactyla**	**Xenarthra/afroteria**	**Chiroptera**	**Artiodactyla a**	**Artiodactyla b**	**Carnivora**	**Marsupialia**	**Prototheria**
	Primata		1.094 ± 0.154	1.107 ± 0.150	1.151 ± 0.164	1.042 ± 0.148	0.833 ± 0.114	0.910 ± 0.141	0.535 ± 0.063	0.480 ± 0.091
	Perissodactyla			1.053 ± 0.170	1.107 ± 0.157	1.006 ± 0.140	0.769 ± 0.094	0.838 ± 0.141	0.505 ± 0.060	0.485 ± 0.072
	Carnivora				1.095 ± 0.168	0.973 ± 0.146	0.856 ± 0.121	0.811 ± 0.129	0.559 ± 0.071	0.500 ± 0.089
	Xenarthra/afroteria					1.041 ± 0.150	0.870 ± 0.136	0.870 ± 0.152	0.517 ± 0.071	0.471 ± 0.083
	Chiroptera						0.877 ± 0.127	0.817 ± 0.130	0.545 ± 0.068	0.494 ± 0.084
	Artiodactyla a							0.620 ± 0.094	0.446 ± 0.042	0.435 ± 0.071
	Artiodactyla b								0.481 ± 0.069	0.429 ± 0.077
	Marsupialia									0.529 ± 0.084
	Prototheria									
**C**		**Primata**	**Perissodactyla**	**Xenarthra/afroteria**	**Chiroptera**	**Artiodactyla a**	**Artiodactyla b**	**Carnivora**	**Marsupialia**	**Prototheria**
	Primata		1.434 ± 0.062	1.449 ± 0.063	1.462 ± 0.026	1.472 ± 0.081	1.461 ± 0.041	1.483 ± 0065	1.548 ± 0.075	1.582 ± 0.047
	Perissodactyla			1.468 ± 0.110	1.452 ± 0.081	1.477 ± 0.084	1.466 ± 0.033	1.509 ± 0.082	1.589 ± 0.085	1.596 ± 0.051
	Xenarthra/afroteria				1.465 ± 0.052	1.480 ± 0.050	1.456 ± 0.036	1.517 ± 0.047	1.569 ± 0.067	1.583 ± 0.036
	Chiroptera					1.474 ± 0.066	1.476 ± 0.039	1.534 ± 0.089	1.574 ± 0.053	1.605 ± 0.038
	Artiodactyla a						1.461 ± 0.039	1.518 ± 0.023	1.579 ± 0.041	1.590 ± 0.045
	Artiodactyla b							1.535 ± 0.029	1.542 ± 0.050	1.579 ± 0.030
	Carnivora								1.568 ± 0.072	1.601 ± 0.037
	Marsupialia									1.600 ± 0.051
	Prototheria									

*Number strings were generated for the Osteopontin sequences under study according to their volume, hydropathy index, solubility, octanol interface, and pI at 25°C. Readouts for similarity were calculated from the strings of numbers representing each property. Then, the resulting values for each physico-chemical characteristic were averaged. Shown are mean ± standard deviation as estimates for evolutionary relatedness.*

***(A)** Autocorrelation averaged across five physico-chemical property scores. For all properties, autocorrelation was compared pairwise between all taxa (the individual results are shown in Supplementary Table S2A). Their averaged numbers are displayed in the table.*

***(B)** Average mutual information across five properties. Average mutual information values were assessed in pairwise comparisons between the number strings representing various properties (the individual results are shown in Supplementary Table S2B). Their averaged numbers are displayed in the table.*

***(C)** Box counting dimension averaged across three properties. The number strings for pairs of taxa were plotted against each other and the resulting fractal dimension was approximated by box counting on a 10 × 10 grid. The box counting dimensions were calculated for isoelectric points, volumes, and solubilities (individual results are shown in Supplementary Table S2C). Their averaged numbers are displayed in the table.*

The average mutual information is information shared between the measurements of two strings. For protein sequences, the average mutual information reflects evolutionary relatedness. We calculated the average mutual information by pairwise comparison between all taxa for each of the physico-chemical parameters under study (Supplementary Table 2B). We averaged the calculated numbers across all five properties (Table 2B).

On a graph that plots the number values for Osteopontins by two species against each other, a close relatedness between the two taxa is reflected in a data distribution near the 45° angle, resulting in a small fractal dimension. More distant relationships are characterized by wider scatter and higher fractal dimensions (practically approximated by the box counting dimension with values between 1 and 2). We calculated the box counting dimensions for the amino acid volumes, isoelectric points and solubilities among all pairs of taxa in this study (Supplementary Table 2C) and utilized their averages as estimates for evolutionary distance (Table 2C).

Once the amino acid sequences of proteins are converted to numerical values that represent their measurable characteristics, those strings become amenable to quantitative evaluation of their similarities. Depending on the focus of the investigation, the properties can be analyzed individually or together. Here, we chose to average autocorrelation, average mutual information, and fractal dimension across all properties evaluated. We interpret the resulting numbers as being reflective of evolutionary distance. Thus, the overall relatedness among protein sequences can be estimated quantitatively by tabulating the values for the above measures (Supplementary Table 3). Of note, the evolutionary distances inferred from autocorrelation, average mutual information and box counting dimension are very similar. The approach is corroborated by the consistent results. It is further validated by the analysis of the subset of avian Osteopontins (Supplementary Figure 6) as well as by the analysis of a different protein, VEGF (Supplementary Figure 7).

### Regional Mutability

**FIGURE 2:**
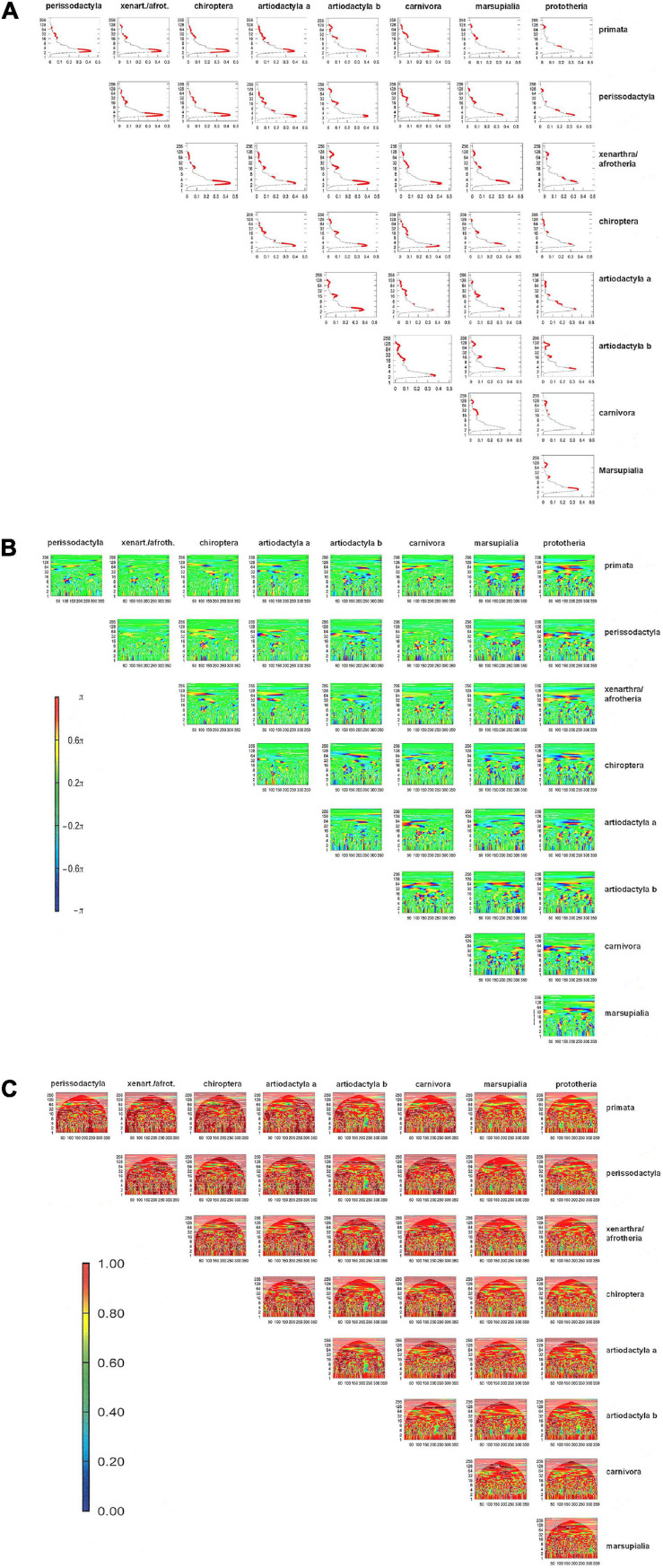
Bivariate wavelet analysis for Osteopontin mutability according to amino acid solubility. Within each subfigure, all comparisons have identical scales for *x*-axis, *y*-axis, and–where indicated–for color intensity. **(A)** Average power plot for amino acid volume. The *x*-axis (0–0.5) indicates the average cross-wavelet power, the *y*-axis represents the length of the Fourier period for synchrony. The solid red dots depict significant joint periods at a probability of error of 0.1. **(B)** Phase difference image. The *x*-axis is reflective of the amino acid position in the protein (1–356), the *y*-axis shows the length of the Fourier period for synchrony (0–256). The color range (π/2,π) and (–π, –π/2) covers shades of blue and red to code for out-of-phase and (–π/2, π/2) shades of green and yellow for in-phase. **(C)** Wavelet coherence plot. The *x*-axis indicates the amino acid position in the protein (1–356), while the *y*-axis displays the length of the Fourier period for synchrony. The color legend shows the magnitude of coherence in frequencies between two sequences, with 1 as high coherence (orange) and 0 low coherence (blue). White contour lines depict significance for joint periodicity, black arrows depict the phase difference in the areas with significant joint periods.

The values pertaining to the physico-chemical characteristics of individual amino acids can be plotted, with the mean values across taxa and a measure of their scatter, on a graph of property (here the values from Table 1) versus position (here amino acid 1 through 356). Domains with no or small error bars are highly conserved, whereas data clusters with large error bars indicate mutable regions (Supplementary Figure 3). In a more stringent approach, the numerical sequences that represent the amino acid properties in each position within a protein can be viewed as waves (which start at position 1 and extend through the length of the protein), Algorithms are available for the comparisons of such waves. Preserved regions display synchrony between the waves that represent two taxa, while mutated regions are visualized as divergence between the waves. Bivariate wavelet analysis can thus reveal domains of evolutionary stability (preserved domains) versus evolutionary instability (Figure 2 and Supplementary Figure 4).

One representation is the cross-wavelet average power plot, which provides a summarized view on the shared periods, the corresponding average power and the associated statistical significance (Figure 2A). Significant joint periods (red dots in Figure 2A) are reflective of low mutability and have higher abundance between species of close evolutionary relatedness.

The phase difference image (Figure 2B) provides a global view of the synchrony status by the two variables at all amino acid positions with different Fourier periods. The graph displays in-phase relationships between the two wavelets as shades of green to yellow, with the *x*-axis indicating the amino acid position and the *y*-axis indicating the corresponding Fourier period. The color shades of green to yellow represent regions of high conservation between the two species under study. The graph also shows out-of-phase relationships as shades of blue and red, and those are regions in the protein with evolutionary instability. Marsupials and prototheria are quite distant from each other and from the other taxa studied here, which is reflected in a high abundance of blue to red areas in the graph.

Two waves are coherent if they have a constant relative phase. The wavelet coherence plot is a measure similar to correlation. It has a value range between 0 and 1, and it shows statistical significance only in areas where the two series being compared actually share significant joint periods (Figure 2C). Coherence is high (red color in the graph, enclosure by white contour lines) between taxa with evolutionary closeness. Green to blue areas in the graph are a readout for the extent of the loss of coherence between two taxa. Their x coordinates show the region of mutability on the protein, while their y coordinates (Fourier period for synchrony) reflect the extent of the difference between taxa. Artiodactyla b display a region of difference from all other taxa between positions 200 and 250. This equates to a stretch of gaps downstream of the RGD motif, where all other taxa have amino acids. Similarly, a stretch of gaps close to position 100 in marsupialia and prototheria is recognizable in the coherence plots.

## Discussion

Existing sequence alignment algorithms use heuristic scoring schemes based on biological expertise, which cannot be used as objective distance metrics (Penner et al., 2011). Conventional phylogenetic trees, based on the alignments of nucleotides or amino acids, often tabulate the numbers of mismatches as a basis for calculating relatedness. Accounting for gaps often is problematic [in a prior phylogenetic analysis of Osteopontin using a large set of sequences (Weber, 2018), the exclusion of gaps caused a substantial reduction in the number of residues that contributed to the calculations]. Further, this approach may place two species at identical distance from all others based on identical numbers of mutated positions (see the conventional phylogenetic trees in Supplementary Figure 2). Trees based on numerical values, which measure amino acid properties, can use the value 0 to account for a gap. Due to the applicable range of such values for the individual amino acids, they are extremely unlikely to encounter identical distances between any pairs. The use of such numbers in tree construction considers not only mutation but also selection as forces of evolution, because selection favors certain mutations over others, and this preference is reflected in the physico-chemical properties of the mutated and selected amino acids (it is not reflected in the assessment of mismatches alone). The resultant tree diagrams are more likely to be a representation of the actual evolutionary distances.

In addition, we note that the calculated values for overall relatedness, comprising autocorrelation, average mutual information, and fractal dimension are interpretable as evolutionary distances that can be mapped in a tree (Supplementary Figure 5). Their use could substantially simplify and unify the construction of phylogenetic trees compared to a diversity of algorithms that are currently in use.

Some techniques of complex systems analysis have been successfully applied to phylogenetic studies, albeit not on the basis of physico-chemical quantities. The patterns of inheritance predict greater similarity in the tempo of molecular evolution between direct ancestors and descendants than in branched relationships, resulting in autocorrelation of evolutionary rates in the tree of life. Rate autocorrelation has turned out to be a common phenomenon throughout evolution (Gillespie, 1984; Tao et al., 2019). Various calculations of mutual information-based distances have yielded better results than conventional distances in distance-based phylogeny. A version based on single letter Shannon entropies gave superior results throughout the entire animal kingdom (Penner et al., 2011). It is reasonable to propose that the logical next step of replacing amino acid identities with their quantifiable properties results in additional improvement.

For the analysis of the highly developed organisms, our approach has produced similar, but not identical trees depending on which parameter was used for generating them. This is not a concern, not only because there is room for improvement of the very basic algorithm applied (as described in section “Methods”). Even for the simple alignment of amino acid sequences followed by counting the number of mismatches, there are diverse methods that yield similar but distinct results [for Osteopontin see Weber (2018), also Supplementary Figure 2]. We believe that the basis of assigned values for various characteristics and their comprehensive analysis offer a path to closer approximation of the actual evolutionary history than the reliance on sequence information alone. Alternatively, for certain proteins, some physico-chemical characteristics may be more important for function than others. It is meaningful to assume that their selection offers a more accurate estimation of the evolutionary history than properties of lesser functional importance. Further, it is noteworthy that calculations of mutual information can assist in judging the relative merits of distinct algorithms by estimating the significance values of specific alignments (Penner et al., 2011). This enables an objective comparison among methods.

This investigation has focused on the innovation of replacing the letter strings with quantitative properties in evolutionary analysis. Further refinements are possible. The box counting procedure is subject to error arising from arbitrary grid placement (quantization error), which is strictly positive and varies as a function of scale. This causes problems for the procedure’s slope estimation step. The quantization error, due to both grid position and orientation, can be a substantial source of error in the estimation of fractal dimension, but pattern search provides an efficient means of minimizing it (Bouda et al., 2016). Future quantitative evolutionary studies, which include the calculation of box counting dimensions, should incorporate such measures.

A wide range of phenomena in nature is describable through strings of numbers. Once a phenomenon has been converted in this manner, the analytical tools of complex systems research become applicable (Abarbanel, 1995) and can yield a plethora of information. Here, we demonstrate the usefulness of converting protein sequence information into numbers that reflect the physico-chemical properties of the constituent amino acids, followed by utilizing this description for close-to-quantitative evolutionary investigation.

## Data Availability Statement

The original contributions presented in the study are included in the article/Supplementary Material, further inquiries can be directed to the corresponding author/s.

## Author Contributions

GW: conceptualization, data analysis, and manuscript preparation. XW: data analysis and manuscript preparation. Both authors contributed to the article and approved the submitted version.

## Conflict of Interest

The authors declare that the research was conducted in the absence of any commercial or financial relationships that could be construed as a potential conflict of interest.

## Publisher’s Note

All claims expressed in this article are solely those of the authors and do not necessarily represent those of their affiliated organizations, or those of the publisher, the editors and the reviewers. Any product that may be evaluated in this article, or claim that may be made by its manufacturer, is not guaranteed or endorsed by the publisher.
