# Supplementary material for: Quantitative Analysis of Protein Evolution: The Phylogeny of Osteopontin
Source: Front Genet. 2021 Aug 16;12:700789. doi: 10.3389/fgene.2021.700789 (PMC8415472; doi:10.3389/fgene.2021.700789)
Supplement: Supplementary file 1 [file Data_Sheet_1.PDF]

Supplement

Table S1: Alignments of the protein sequences for Osteopontin by various taxa. The sequences were aligned with Clustal Omega by EMBL-EBI at the default settings.

Table S2A: Autocorrelation for the individual properties under study. Displayed are the tables for the autocorrelation values, calculated pairwise between the Osteopontin protein sequences belonging to various taxa. Each table indicates one of the physico-chemical properties that were used to convert the amino acid letter strings to numerical sequences.

Table S2B: Average mutual information for the individual properties under study. Displayed are the tables for the calculated values, pairwise between the Osteopontin protein sequences belonging to various taxa. Each table indicates one of the physico-chemical properties that were used to convert the amino acid letter strings to numerical sequences.

Table S2C: Box counting dimension for various properties under study. Graphs plotted the numbers for the properties by all taxa pairwise against each other to evaluate for the resulting box counting dimensions (which approximate their fractal dimensions). Each table indicates one of the physico-chemical properties that were used to convert the amino acid letter strings to numerical sequences. Top = isoelectric point (pI), middle = volume, bottom = solubility.

Table S3: Color-coded tables comparing evolutionary relatedness as calculated from various measurements. Displayed are 1/autocorrelation, 1/average mutual information (top and middle; each averaged from volume, hydropathy index, solubility, octanol interface, and pI at 25°C), and fractal dimension (bottom; averaged from isoelectric points, volumes, and solubilities). The lowest value, indicative of closest relatedness, has yellow background. The highest value, indicative of large evolutionary distance, is shown on green background. The shades in between represent the range.

Figure S1: Number strings for solubility. In the consensus sequences, the letters representing individual amino acids were replaced by their values for solubility (g/kg H_2O). The graphs show pairwise comparisons of the taxa under investigation for solubility (y-axis) versus amino acid position (x-axis).

Figure S2: Conventional phylogenetic trees for Osteopontin. A) phylogeny. Tree generated in phylogeny.fr (http://www.phylogeny.fr/). **B) TreeTop.** Tree generated with the TreeTop function in Gene Bee (http://www.genebee.msu.su/services/phtree_reduced.html).

Figure S3: Regional mutability of Osteopontin. Shown are mean values and standard deviations across all nine taxa for each property under study (volume, solubility, isoelectric point, octanol interface, and hydropathy) at each position in the protein (1-356). On the bottom, three regions with high variation throughout evolution are indicated by horizontal red bars. They correspond to positions 95-125 (poly-aspartate), 198-246, and 299-329. Because these regions are characterized by gaps/insertions, a value of 0 is common to a fraction of the taxa.

Figure S4: Bivariate wavelet analysis related to various physico-chemical properties. Displayed are the graphs for average cross-wavelet power (A, D, G, J), phase difference (B, E, H, K), and cross-wavelet coherence (C, F, I, L). **A-C) Hydropathy. D-F) Octanol interface. G-I) Isoelectric point. J-L) Amino acid volume.**

Figure S5: Phylogenetic tree for Osteopontin evolution. Based on the average mutual information (mean values across all physico-chemical properties, as shown in Table S3), a phylogenetic tree was generated with the stepwise algorithm described in Methods. The two taxa with the smallest 1/(average mutual information) at each step were combined and their values were averaged before repeating the process. The numbers shown at the branches express the distances as calculated, the lengths of the lines are drawn to scale (they are proportional to the smallest 1/(average mutual information) calculated at each step).

Figure S6: Phylogenetic analysis for avian Osteopontin. A) Sequence alignments. Canonical sequences were inferred for each of the four clades previously identified, followed by alignment with Clustal Omega by EMBL-EBI at the default settings. **B) Conventional phylogenetic trees.** (top) Tree generated in phylogeny.fr (http://www.phylogeny.fr/)**.** (Bottom) Tree generated with the TreeTop function in Gene Bee (http://www.genebee.msu.su/services/phtree_reduced.html). **C) Quantitative phylogenetic analysis.** The amino acid sequences were converted to strings of numbers reflecting their physico-chemical properties. Phylogenetic trees were generated on the basis of the sum differences between strings, such that the strings with the smallest sum-difference at each step were averaged before repeating the process. The numbers express the distances as calculated

from the indicated properties, the lengths of the lines are drawn to scale (they are proportional to the numerical differences calculated). (Bottom right) Based on the autocorrelation (mean values across all physico-chemical properties, as shown in D)), a phylogenetic tree was generated with the stepwise algorithm described in Methods. **D) Autocorrelation for the individual properties under study.** Displayed are the tables for the autocorrelation values, calculated pairwise between the Osteopontin protein sequences belonging to the individual clades of birds. Each table indicates one of the physico-chemical properties that were used to convert the amino acid letter strings to numerical sequences. The bottom table displays the average over all properties studied.

Figure S7: Phylogenetic analysis of Vascular Endothelial Growth Factor. The vascular endothelial growth factor (VEGF) coding sequences were retrieved from Homo sapiens, Piliocolobus tephrosceles, Sus scrofa, Spalax ehrenbergi, Xenopus laevis, Passer montanus, Gallus gallus, Trimeresurus flavoviridis, Bovine papular stomatitis virus strain V660, Parapoxvirus of red deer strain RD86. **A) Sequence alignments.** The selected sequences were aligned with Clustal Omega by EMBL-EBI at the default settings. **B) Regional mutability of VEGF.** Shown are mean values and standard deviations across all species for each property under study (hydropathy, molecular weight, octanol interface, isoelectric point, solubility) at each position in the protein (1-230). **C) Autocorrelation for the individual properties under study.** Displayed are the tables for the autocorrelation values, calculated pairwise between the VEGF protein sequences belonging to the selected species. Each table indicates one of the physico-chemical properties that were used to convert the amino acid letter strings to numerical sequences. The bottom table displays the average over all properties studied. **D) Average mutual information across five properties.** Average mutual information values were assessed in pairwise comparisons between the number strings representing each of the indicated properties. Their averaged numbers are displayed in the bottom Table. **E) Return plots.** Octanol interface values of the human sequence (homo, x-axis) are compared pairwise to each of the other selected species (y-axis). While there is almost complete identity to the monkey sequence (pilocolobus), reflected in the distribution close to the identity line of 45° angle, the scatter widens with increasing evolutionary distance. **F-N) Bivariate wavelet analysis related to various physico-chemical properties.** Displayed are the graphs for average cross-wavelet power (F, I, L), phase difference (G, J, M), and cross-wavelet coherence (H, K, N). **F-H) Hydropathy. I-K) Octanol interface. L-N) Isoelectric point.**

Table S1

```
CLUSTAL O(1.2.4) multiple sequence alignment


prototheria              MRTAVICLCLISIACALPVKRSGPEANSGSSEEKQLYNKHPNQLSSWLNTDPSQKQALLA  60
marsupialia              MRTAVICFCLLGIVSALPVKQ---QINSGSSEEKRLYSKHPNFVATWLNADPSQKQTLLA  57
carnivora                MRIAVICFCLLGIAYTIPIKQ----TDSGSSEEKQLYNKYPGAVATWPKPDPSQKQTFLA  56
artiodactyla(b)          MRIAVICFCLLGIASALPVKP----TSSGSSEEKQLNNKYPDAVATWLKPDPSQKQTFLA  56
artiodactyla(a)          MKIAVICFCLLGIASALPVGQ----TDSGSSEEKQLNNKYPDAVATWLKPDPSQKQTFLA  56
perissodactyla           MRIAVICLCLLGIAYALPVNQ----ADSGSSEEKQLYNKHSDAVSIWLKPDPSQKQNLLA  56
afroteria,xenarthra      MRIAVICFCLLGIAYALPVKH---QADSGSSEEKQLYSKYPDAVATWLKPDPSYKQILLA  57
chiroptera               MRIAVICFCLLGIAYALPVKH----ADSGSSEERQLYNKHPDAVATWLKPDPSQKQSLLA  56
primata                  MRIAVICFCLLGIAYALPVKQ----ADSGSSEEKQLYNKYPDAVATWLKPDPSQKQNLLA  56
                         *: ****:**:.*. ::*:       .******::* .*: . :: * : *** ** :**  60


prototheria              PQNLVSSEESKENIQQQTLPSISNESHDDVDDADDQDDSD--------------------  100
marsupialia              TQNSVSSEESTE-DLQETLPSNSNESPDDIDDVDDDDGDEGH------------------  98
carnivora                LQNAVLSEETDD-FKQKTLASKSNESHDDVDEDD----EDDVDSQDSVDSHD--------  103
artiodactyla(b)          PQNSVSSEETDD-NKQNTLPSKSNESPEQTDDLDDDDDNSQ-----DVTSDDSDDADNPD  110
artiodactyla(a)          PQSTASSEETDD-NKQETLPSTSNESPDHTDDVDDDDDDDVDD----QDVDSNDSDDHTD  111
perissodactyla           PQT-VSSEETDN-LKQETLPSQSNESHDHTDDVDDDDVGDHEDDQDSIDSDDSDETDPTD  114
afroteria,xenarthra      PQNAVSSEETDH-LKQETLPSKSNESHDQTDDVDDEDDGDHVDSQDSVDSDDSDDDDHTD  116
chiroptera               PQNAVSSEETDD-FKQETLPSQSNESQDLTDDVDDDDDEDHVDSQDSIDSNDSDDADHTD  115
primata                  PQNAVSSEETND-FKQETLPSKSNESHDHMDDVDDEDDDDHVDSQDSIDSNDSDEVDDTD  115
                          *. . ***: .   *:** * **** :  *: *     .                      120


prototheria              ---HKDESDDSDESDEVVTDFPTDVPATAVFTPAAPTRGDNGGRGDRVYRGLKTKPGVLY  157
marsupialia              ---KSTDSDDSDESDEVVTDFPTDTPATSSFLPDGPTRGDNGGRGDSVAYGLRSKVGAPY  155
carnivora                ---TDDDSNQSDESDELVTDFPTDVPATQFFTPAVPTRDSYDGRGDSVAYGLRSKSKKSH  160
artiodactyla(b)          DPDHSDESHHSDESDEV--DFPTDIPTTAVSTPPIPTENTNDGRGDSVAYGLKSKSKKFR  168
artiodactyla(a)          DTDRSDESHHSDESDEVVTDFPTDIPATAVFTPAVPTEDTQDGRGDNVAYALRTKSKKFR  171
perissodactyla           DPDNSDESHHSDESDELVTDFSTDVPATPVFTPAVPTRDTYDGRGDSLSYGLKSKSRKFR  174
afroteria,xenarthra      DPDHSDESHHSDESDELVTDFPTDDPATPVFTPAVPTVDTYDGRGDSVVYRLRSKSKKIH  176
chiroptera               DSDNSDESHHSDESDELVTDFPTDFPGTPFATPAVPTRDTNDGRGDSVAYRLKLKSKKFH  175
primata                  DSHQSDESHHSDESDELVTDFPTDLPATEVFTPVVPTVDIYDGRGDSVAYGLRSKSKKFR  175
                             . :*..******:  ** ** * *    *  ** .  .**** :   *: *       180
```

```
prototheria             KAAVQGHDAS-DDFTSRLESLESDES---PEAYPDAHKLQKSSEWHSNEASHQDDRSMQK  213
marsupialia             RSSEQVHDVTEEDLTSQMESYESEKA---HKAFPLSQNLPKVSAWGSNSK----------  202
carnivora               RYEDQYPDSTEEDFTSLVKSESMEDD---FNAVLLSHTVRGSSDRDSHVK----------  207
artiodactyla(b)         RSSVQSPDATEEDFTSHVESEEMHDAP---------------------------------  195
artiodactyla(a)         RSEVQHPDATEEDVTSHVESEEADDA---PKAILVAQRLHRASDWDSRGK----------  218
perissodactyla          RSEDQYPDATEEDLTSPVESKDIDDV---HKAVLVAQGLHVASDWDSRGK----------  221
afroteria,xenarthra     RSVIQFPDATEEDLTSQMESKELDDA---HKAIPVIQSLKKVSDWDSQQQ----------  223
chiroptera              RSAVQYPDATEEDLTSHVESKEMDDARKAVKAVLVAQGLPVASDWDSHGK----------  225
primata                 RPDIQYPDATDEDITSHVESEELNGA---YKAIPVAQGLNVPSDWDSRGK----------  222
                        :   *  * : :*.** ::* . .                                      240

prototheria             SSEWHSSEASHQDDRSVETHSHEEAKGYRLKQED-HSSQQDDLNDSQESYKVSRENDSQE  272
marsupialia             ----ESNEASHPDEYSVETYSHEQLKSYQLERNIYDSQQQSDSHGSQENDKVSQEFHNRE  258
carnivora               ----DSQETSQVDDHSMETKSRRHSKEYKLKASD-ENNKHSHEIGSQESSDVSSELVGQT  262
artiodactyla(b)         ------KKTSQLNDHSKETNSNELSKELKPKAKD-E-SKHSDVIESQENSKVSQEFHS--  245
artiodactyla(a)         ----DSQETSQPDDRSMETHSREHSEEFKLKAED-ESDRHSDVIDSQDNSKVSPEVHSQE  273
perissodactyla          ----DSQETSQLDDHSVETHSREHSKEYQLKAND-ETSEHSDVIDSRENSKVSQEFPSQE  276
afroteria,xenarthra     ----DSHETSQPDDHSVETHSQEQSKEYKLKAND-VSSEHSDTIDSQENSKVSQEFHS--  276
chiroptera              ----DSQETSQLDDHSTETHSLKLAKEYKLKAND-ASNGHSDVIDSQENSKVSHEFHSRE  280
primata                 ----DSHETSQLDDQSAETHSHKQSRLYKRKASD-DSNEHSDVIDSQERSKVSREFHSHE  277
                               ::*: :: * ** * .  .  : : .    . :..   *::  .** *  .    300

prototheria             KTAQ----------------------DADSDEFNRKHYLKSHTSHEFDSASSETH      305
marsupialia             VDRDSQEFHKQQVGKLSQEYHSQEVHLVSDPESVENIKPLKLHSFPEADSASYEAH      314
carnivora               -------------------VQSNEKELVLDSESEEEDKHLKFRVSHELDSASSEVN      299
artiodactyla(b)         ----------------------LEDKLDLDHKS-EEDKHLKIRISHELDSTSSEVN      278
artiodactyla(a)         -------------------LPSHEDKLVLDPKSEEEDTHLKFRVSHELDSASSEVN      310
perissodactyla          -------------------FHSSEGKLVRDRKSEDEDKYLKFRTSHESESASSEVN      313
afroteria,xenarthra     ----------------------HEDKHVPDPKSEEEDKHLKFRTSHELESTSYEIN      310
chiroptera              -------------------LHSREDKLALDPQSEEQDKHLKFRISHELESASSEVN      317
primata                 -------------------FHSHEDMLVVDPKSKEEDKHLKFRISHELDSASSEVN      314
                                                     * .. :.   ** :   * :*:* * :      356
```

Table S2A

hydropathy

	primata	perisso-dactyla	carnivora	xenarthra/afroteria	chiroptera	artiodactyla a	artiodactyla b	marsupialia	prototheria
**primata**		0.822	0.757	0.795	0.857	0.791	0.726	0.555	0.568
**perissodactyla**			0.800	0.799	0.813	0.830	0.815	0.651	0.579
**carnivora**				0.720	0.805	0.746	0.726	0.576	0.535
**xenarthra/afroteria**					0.798	0.787	0.780	0.619	0.666
**chiroptera**						0.830	0.893	0.561	0.566
**artiodactyla a**							0.748	0.638	0.630
**artiodactyla b**								0.590	0.615
**marsupialia**									0.599
**prototheria**									

octanol interface

	primata	perisso-dactyla	carnivora	xenarthra/afroteria	chiroptera	artiodactyla a	artiodactyla b	marsupialia	prototheria
**primata**		0.789	0.703	0.837	0.857	0.807	0.665	0.446	0.512
**perissodactyla**			0.766	0.851	0.841	0.778	0.723	0.516	0.501
**carnivora**				0.741	0.749	0.642	0.602	0.437	0.521
**xenarthra/afroteria**					0.874	0.837	0.779	0.524	0.594
**chiroptera**						0.819	0.818	0.413	0.520
**artiodactyla a**							0.681	0.472	0.549
**artiodactyla b**								0.438	0.561
**marsupialia**									0.566
**prototheria**									

pI (25oC)

	primata	perisso-dactyla	carnivora	xenarthra/afroteria	chiroptera	artiodactyla a	artiodactyla b	marsupialia	prototheria
**primata**		0.836	0.842	0.795	0.822	0.810	0.711	0.513	0.449
**perissodactyla**			0.729	0.846	0.846	0.839	0.573	0.554	0.443
**carnivora**				0.699	0.732	0.770	0.510	0.534	0.474
**xenarthra/afroteria**					0.815	0.788	0.601	0.525	0.423
**chiroptera**						0.767	0.556	0.513	0.419
**artiodactyla a**							0.762	0.510	0.476
**artiodactyla b**								0.311	0.351
**marsupialia**									0.525
**prototheria**									

solubility

	primata	perisso-dactyla	carnivora	xenarthra/afroteria	chiroptera	artiodactyla a	artiodactyla b	marsupialia	prototheria
**primata**		0.849	0.812	0.836	0.859	0.770	0.701	0.603	0.534
**perissodactyla**			0.737	0.797	0.837	0.811	0.754	0.591	0.561
**carnivora**				0.702	0.715	0.726	0.644	0.528	0.516
**xenarthra/afroteria**					0.820	0.755	0.768	0.572	0.693
**chiroptera**						0.804	0.739	0.605	0.545
**artiodactyla a**							0.700	0.645	0.591
**artiodactyla b**								0.590	0.576
**marsupialia**									0.578
**prototheria**									

volume

	primata	perisso-dactyla	carnivora	xenarthra/afroteria	chiroptera	artiodactyla a	artiodactyla b	marsupialia	prototheria
**primata**		0.944	0.903	0.891	0.890	0.888	0.779	0.489	0.435
**perissodactyla**			0.764	0.887	0.919	0.902	0.584	0.472	0.430
**carnivora**				0.732	0.719	0.867	0.569	0.526	0.481
**xenarthra/afroteria**					0.866	0.866	0.622	0.466	0.442
**chiroptera**						0.848	0.584	0.449	0.398
**artiodactyla a**							0.800	0.533	0.494
**artiodactyla b**								0.306	0.323
**marsupialia**									0.474
**prototheria**									

Table S2B

volume

	primata	perisso-dactyla	xenarthra/ afroteria	chiroptera	artiodactyla a	artiodactyla b	carnivora	marsupialia	prototheria
**primata**		1.167	1.116	1.216	1.040	0.848	0.924	0.535	0.466
**perissodactyla**			1.086	1.169	1.001	0.781	0.903	0.504	0.449
**xenarthra.afroteria**				1.096	0.961	0.874	0.803	0.550	0.478
**chiroptera**					1.044	0.866	0.866	0.497	0.435
**artiodactyla.a**						0.898	0.823	0.523	0.464
**artiodactyla.b**							0.645	0.453	0.398
**carnivora**								0.453	0.400
**marsupialia**									0.527
**prototheria**									

hydropathy

	primata	perisso-dactyla	xenarthra/ afroteria	chiroptera	artiodactyla a	artiodactyla b	carnivora	marsupialia	prototheria
**primata**		1.186	1.166	1.226	1.126	0.908	0.984	0.584	0.565
**perissodactyla**			1.088	1.155	1.085	0.833	0.879	0.538	0.564
**xenarthra.afroteria**				1.130	1.027	0.910	0.868	0.597	0.591
**chiroptera**					1.117	0.962	0.941	0.577	0.537
**artiodactyla.a**						0.944	0.889	0.612	0.566
**artiodactyla.b**							0.724	0.476	0.491
**carnivora**								0.557	0.516
**marsupialia**									0.577
**prototheria**									

octanol interface

	primata	perisso-dactyla	xenarthra/ afroteria	chiroptera	artiodactyla a	artiodactyla b	carnivora	marsupialia	prototheria
**primata**		1.178	1.258	1.279	1.149	0.928	1.030	0.605	0.574
**perissodactyla**			1.225	1.271	1.108	0.842	0.959	0.565	0.561
**xenarthra.afroteria**				1.290	1.129	0.942	0.949	0.652	0.588
**chiroptera**					1.183	1.002	1.029	0.592	0.577
**artiodactyla.a**						0.983	0.920	0.618	0.594
**artiodactyla.b**							0.715	0.491	0.520
**carnivora**								0.537	0.497
**marsupialia**									0.620
**prototheria**									

pI (25oC)

	primata	perisso-dactyla	xenarthra/ afroteria	chiroptera	artiodactyla a	artiodactyla b	carnivora	marsupialia	prototheria
**primata**		1.116	1.140	1.167	1.107	0.838	0.947	0.505	0.437
**perissodactyla**			1.098	1.086	1.069	0.781	0.855	0.512	0.446
**xenarthra.afroteria**				1.131	1.014	0.911	0.832	0.527	0.461
**chiroptera**					1.071	0.868	0.891	0.504	0.435
**artiodactyla.a**						0.904	0.862	0.508	0.451
**artiodactyla.b**							0.673	0.425	0.420
**carnivora**								0.470	0.407
**marsupialia**									0.529
**prototheria**									

solubility

	primata	perisso-dactyla	xenarthra/ afroteria	chiroptera	artiodactyla a	artiodactyla b	carnivora	marsupialia	prototheria
**primata**		0.822	0.857	0.866	0.788	0.642	0.668	0.447	0.358
**perissodactyla**			0.768	0.853	0.765	0.610	0.596	0.408	0.408
**xenarthra.afroteria**				0.826	0.736	0.643	0.601	0.466	0.383
**chiroptera**					0.789	0.652	0.622	0.414	0.373
**artiodactyla.a**						0.658	0.593	0.464	0.394
**artiodactyla.b**							0.491	0.386	0.346
**carnivora**								0.386	0.327
**marsupialia**									0.395
**prototheria**									

Table S2C

isoelectric point

	primata	perisso-dactyla	carnivora	xenarthra/afroteria	chiroptera	artiodactyla a	artiodactyla b	marsupialia	prototheria
**primata**		1.398	1.415	1.380	1.431	1.380	1.477	1.491	1.531
**perissodactyla**			1.415	1.342	1.362	1.380	1.447	1.491	1.556
**carnivora**				1.477	1.431	1.491	1.519	1.491	1.580
**xenarthra/afroteria**					1.415	1.431	1.415	1.491	1.556
**chiroptera**						1.398	1.431	1.519	1.568
**artiodactyla a**							1.447	1.544	1.544
**artiodactyla b**								1.491	1.556
**marsupialia**									1.544
**prototheria**									

volume

	primata	perisso-dactyla	carnivora	xenarthra/afroteria	chiroptera	artiodactyla a	artiodactyla b	marsupialia	prototheria
**primata**		1.398	1.544	1.505	1.477	1.505	1.491	1.633	1.623
**peissodactyla**			1.556	1.519	1.477	1.519	1.505	1.633	1.653
**carnivora**				1.568	1.591	1.531	1.568	1.633	1.643
**xenarthra/afroteria**					1.519	1.531	1.477	1.613	1.623
**chiroptera**						1.505	1.505	1.623	1.643
**artiodactyla a**							1.505	1.623	1.633
**artiodactyla b**								1.591	1.613
**marsupialia**									1.643
**protohteria**									

solubility

	primata	perisso-dactyla	carnivora	xenarthra/afroteria	chiroptera	artiodactyla a	artiodactyla b	marsupialia	prototheria
**primata**		1.505	1.491	1.462	1.477	1.531	1.415	1.519	1.591
**perissodactyla**			1.556	1.544	1.519	1.531	1.447	1.643	1.580
**carnivora**				1.505	1.580	1.531	1.519	1.580	1.580
**xenarthra/afroteria**					1.462	1.477	1.477	1.602	1.568
**chiroptera**						1.519	1.491	1.580	1.602
**artiodactyla a**							1.431	1.568	1.591
**artiodactyla b**								1.544	1.568
**marsupialia**									1.613
**prototheria**									

Table S3

1/autocorrelation

	primata	perisso-dactyla	xenarthra/afroteria	chiroptera	artiodactyla a	artiodactyla b	carnivora	marsupialia	prototheria
**primata**		1.179	1.203	1.167	1.230	1.396	1.245	1.919	2.002
**perissodactyla**			1.196	1.175	1.202	1.450	1.318	1.796	1.988
**xenarthra/afroteria**				1.198	1.240	1.408	1.391	1.848	1.774
**chiroptera**					1.229	1.393	1.344	1.968	2.042
**artiodactyla a**						1.355	1.333	1.788	1.825
**artiodactyla b**							1.640	2.237	2.061
**carnivora**								1.922	1.978
**marsupialia**									1.823
**prototheria**									

1/average mutual information

	primata	perisso-dactyla	xenarthra/afroteria	chiroptera	artiodactyla a	artiodactyla b	carnivora	marsupialia	prototheria
**primata**		0.914	0.903	0.869	0.960	1.201	1.098	1.868	2.082
**perissodactyla**			0.950	0.904	0.994	1.300	1.193	1.979	2.060
**xenarthra/afroteria**				0.914	1.027	1.168	1.234	1.790	1.999
**chiroptera**					0.961	1.150	1.150	1.934	2.121
**artiodactyla a**						1.140	1.223	1.834	2.024
**artiodactyla b**							1.539	2.241	2.300
**carnivora**								2.080	2.330
**marsupialia**									1.889
**prototheria**									

fractal dimension

	primata	perisso-dactyla	xenarthra/afroteria	chiroptera	artiodactyla a	artiodactyla b	carnivora	marsupialia	prototheria
**primata**		1.434	1.449	1.462	1.472	1.461	1.483	1.548	1.582
**perissodactyla**			1.468	1.452	1.477	1.466	1.509	1.589	1.596
**xenarthra/afroteria**				1.465	1.480	1.456	1.517	1.569	1.583
**chiroptera**					1.474	1.476	1.534	1.574	1.605
**artiodactyla a**						1.461	1.518	1.579	1.590
**artiodactyla b**							1.535	1.542	1.579
**carnivora**								1.568	1.601
**marsupialia**									1.600
**prototheria**									

Figure S1

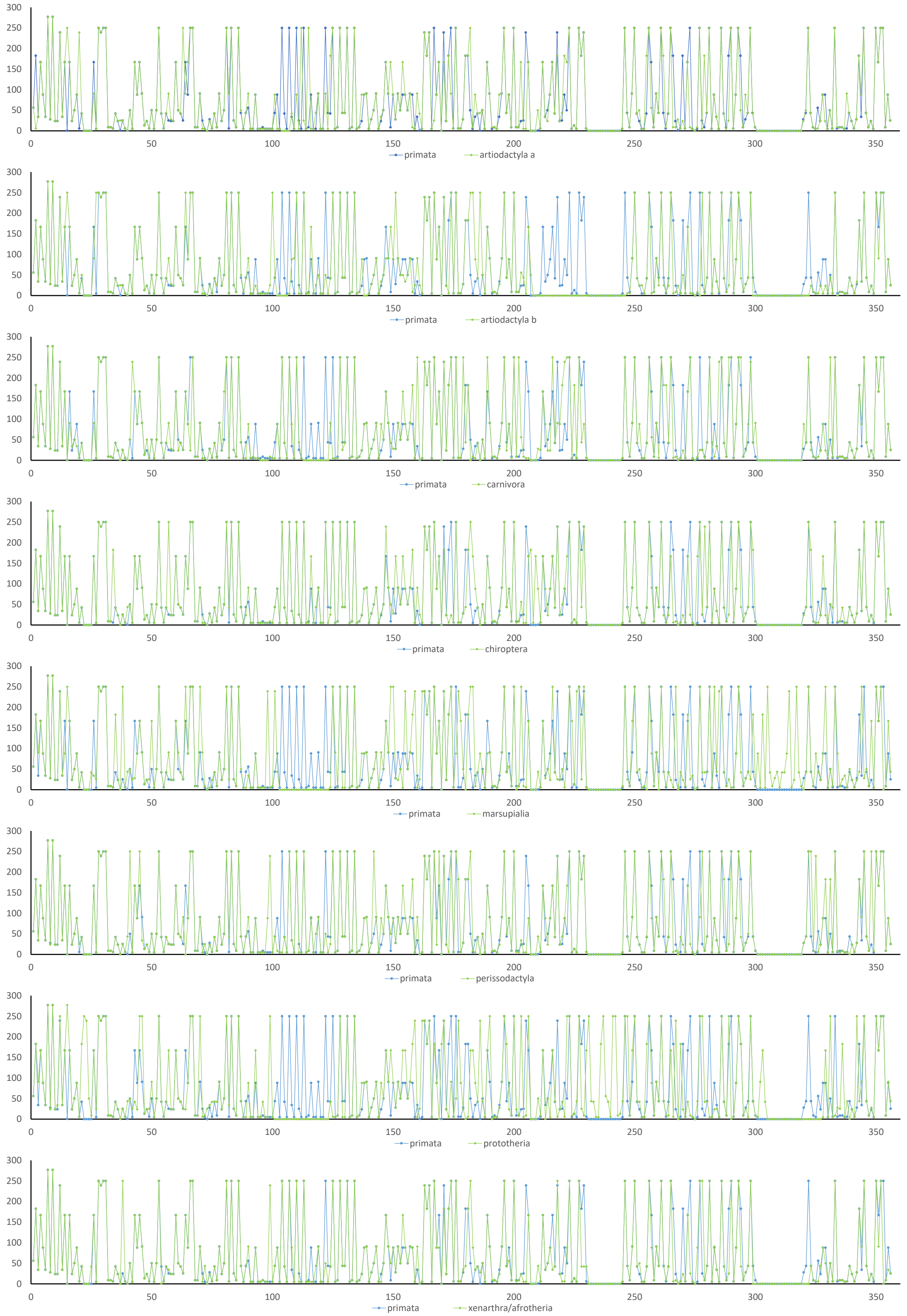
primata
artiodactyla a
primata
artiodactyla b
primata
carnivora
primata
chiroptera
primata
marsupialia
primata
perissodactyla
primata
prototheria
primata
xenarthra/afrotheria

Figure S2

A)

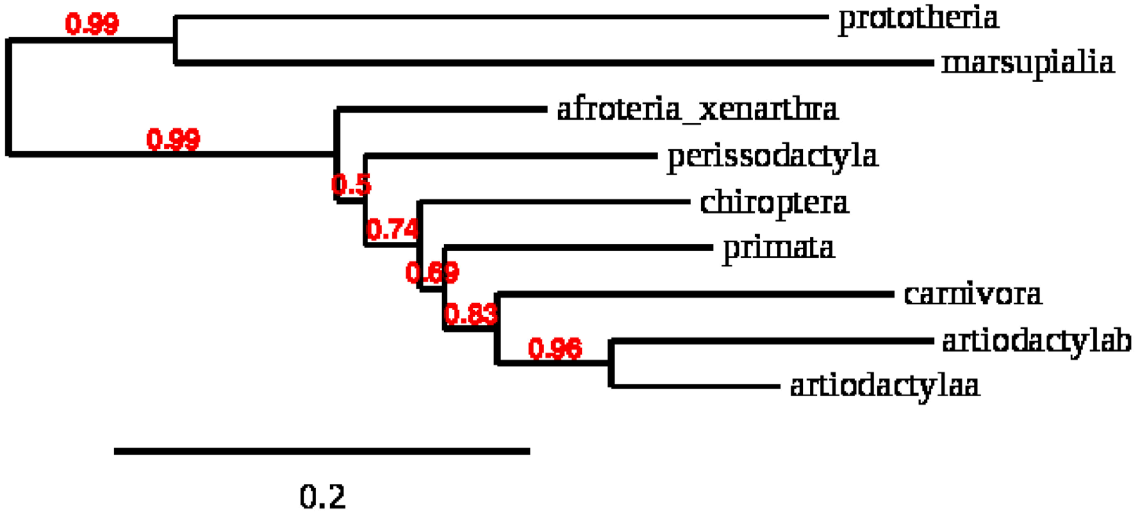
0.99
prototheria
marsupialia
0.99
afroteria_xenarthra
perissodactyla
0.5
chiroptera
0.74
primata
0.69
carnivora
0.83
0.96
artiodactylab
artiodactylaa
0.2

B)

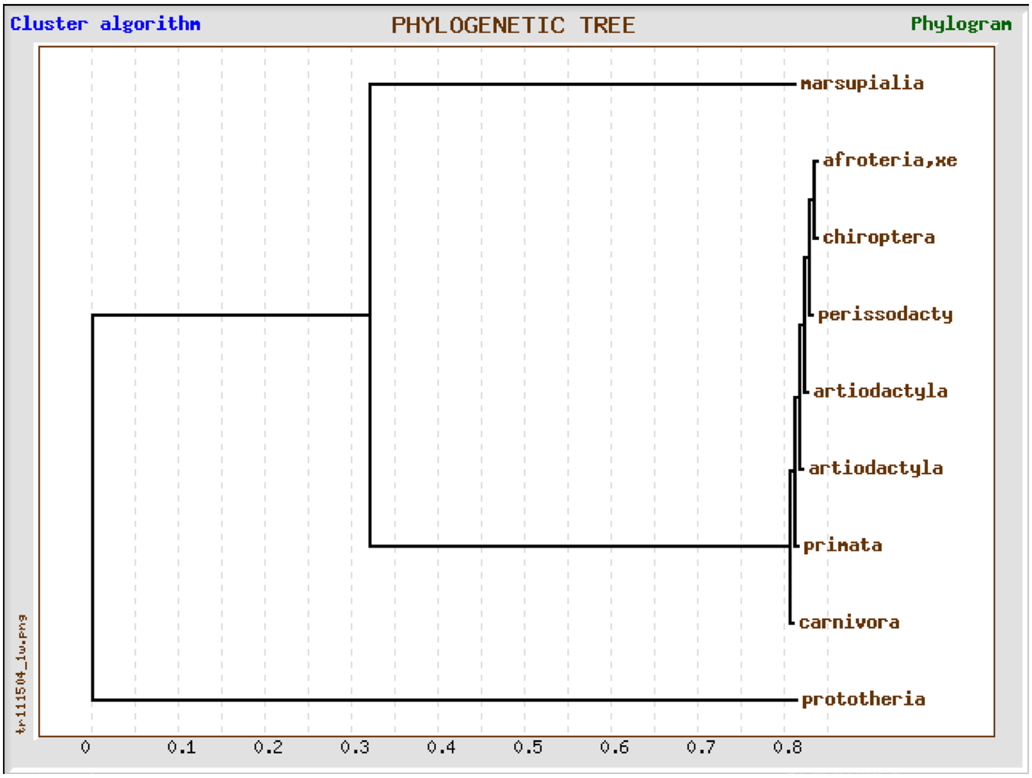
Cluster algorithm
PHYLOGENETIC TREE
Phylogram
marsupialia
afroteria,xe
chiroptera
perissodacty
artiodactyla
artiodactyla
primata
carnivora
prototheria
0
0.1
0.2
0.3
0.4
0.5
0.6
0.7
0.8

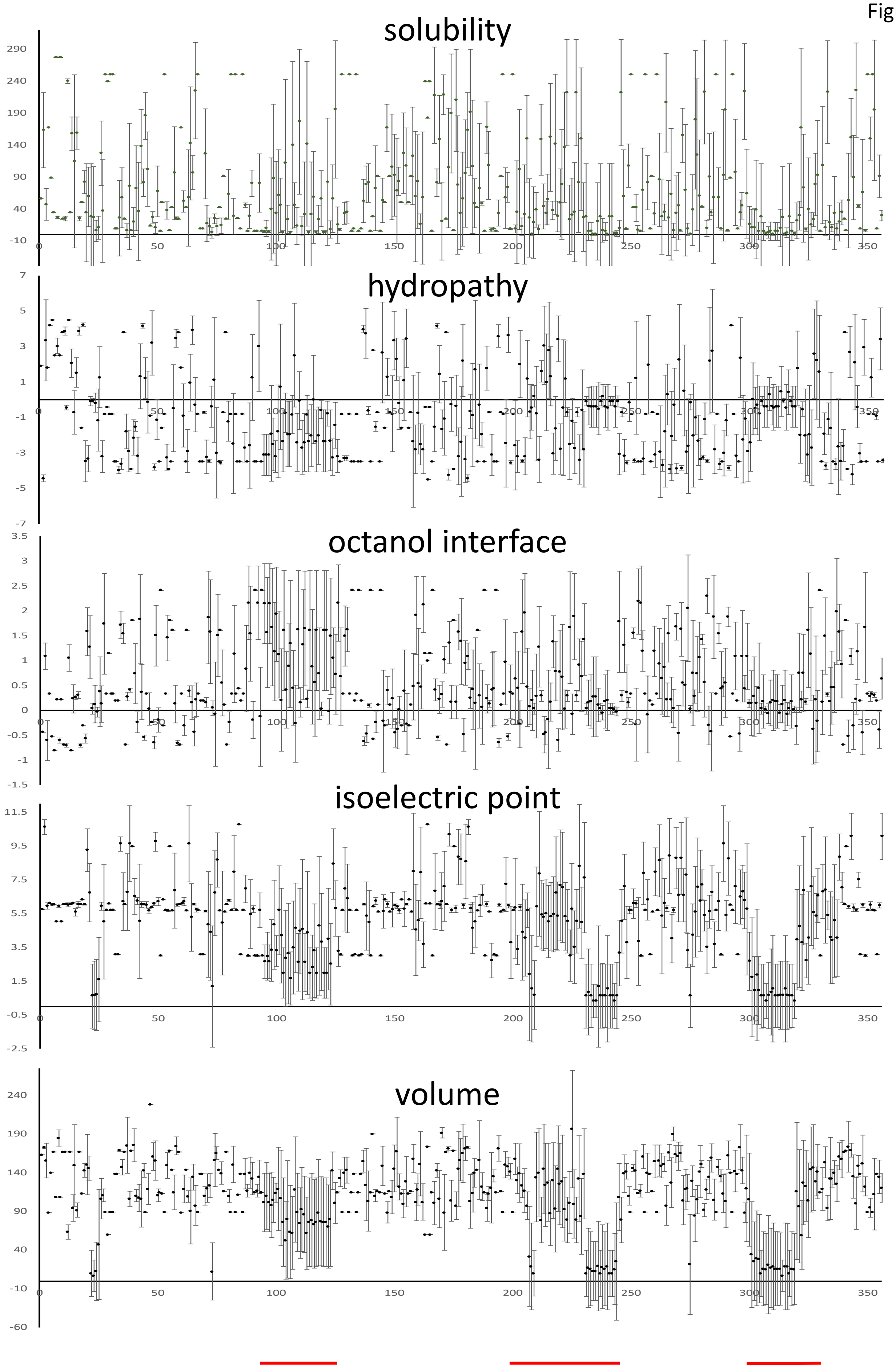
solubility
hydropathy
octanol interface
isoelectric point
volume

Figure S4A

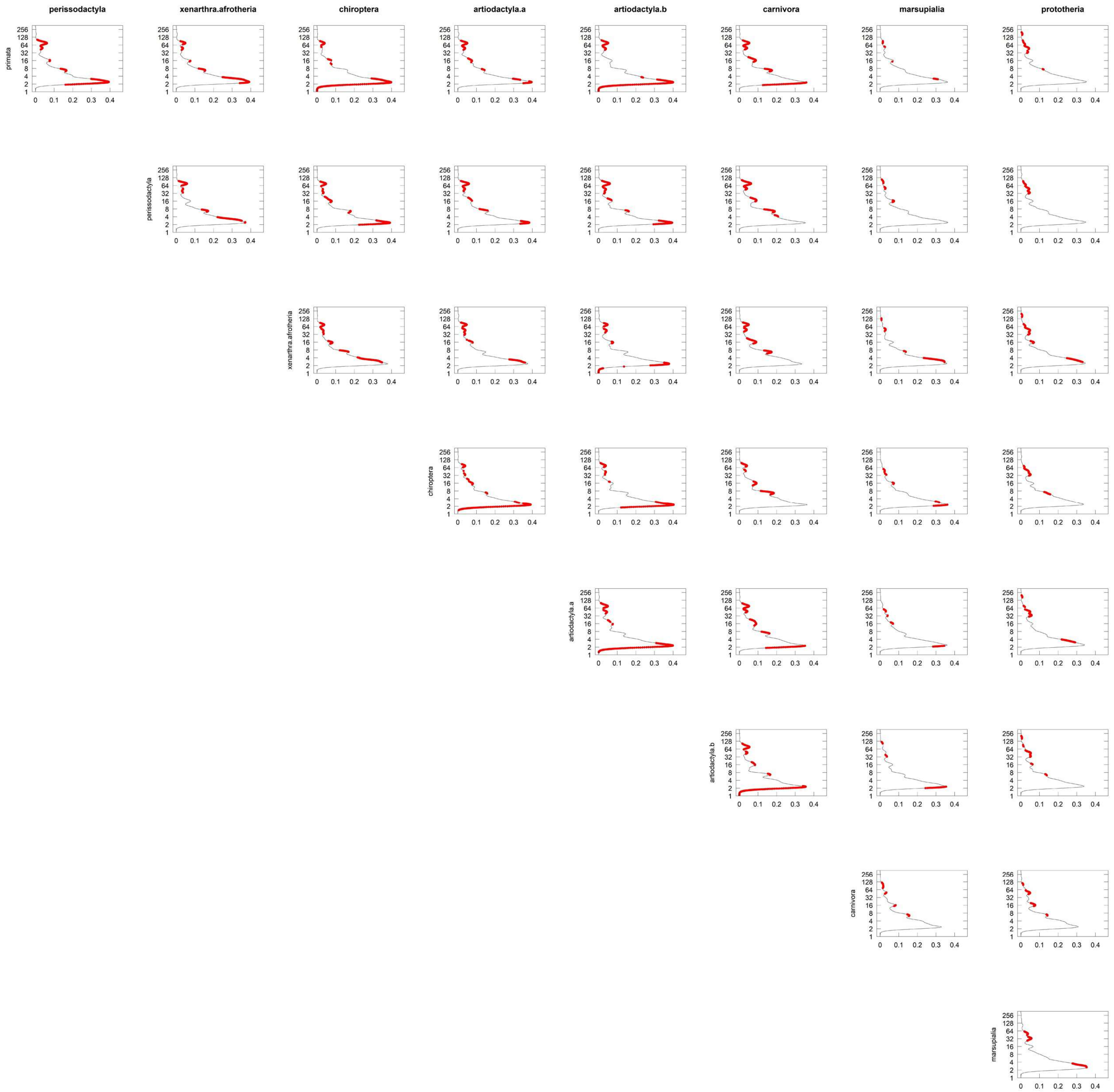
perissodactyla
xenarthra.afrotheria
chiroptera
artiodactyla.a
artiodactyla.b
carnivora
marsupialia
prototheria
primata
perissodactyla
xenarthra.afrotheria
chiroptera
artiodactyla.a
artiodactyla.b
carnivora
marsupialia
256
128
64
32
16
8
4
2
1
0
0.1
0.2
0.3
0.4

Figure S4B

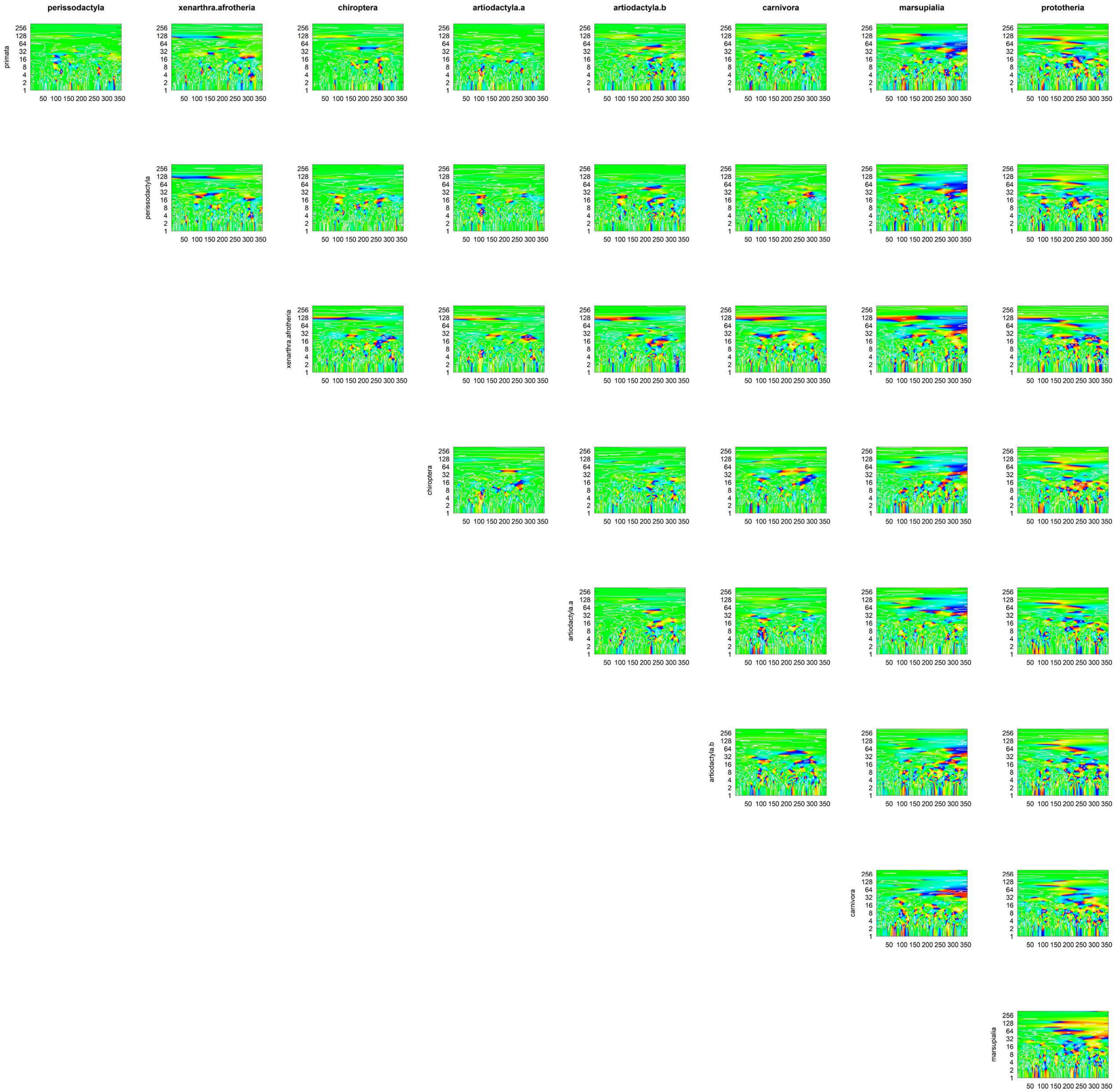
perissodactyla
xenarthra.afrotheria
chiroptera
artiodactyla.a
artiodactyla.b
carnivora
marsupialia
prototheria
primata
perissodactyla
xenarthra.afrotheria
chiroptera
artiodactyla.a
artiodactyla.b
carnivora
marsupialia

Figure S4C

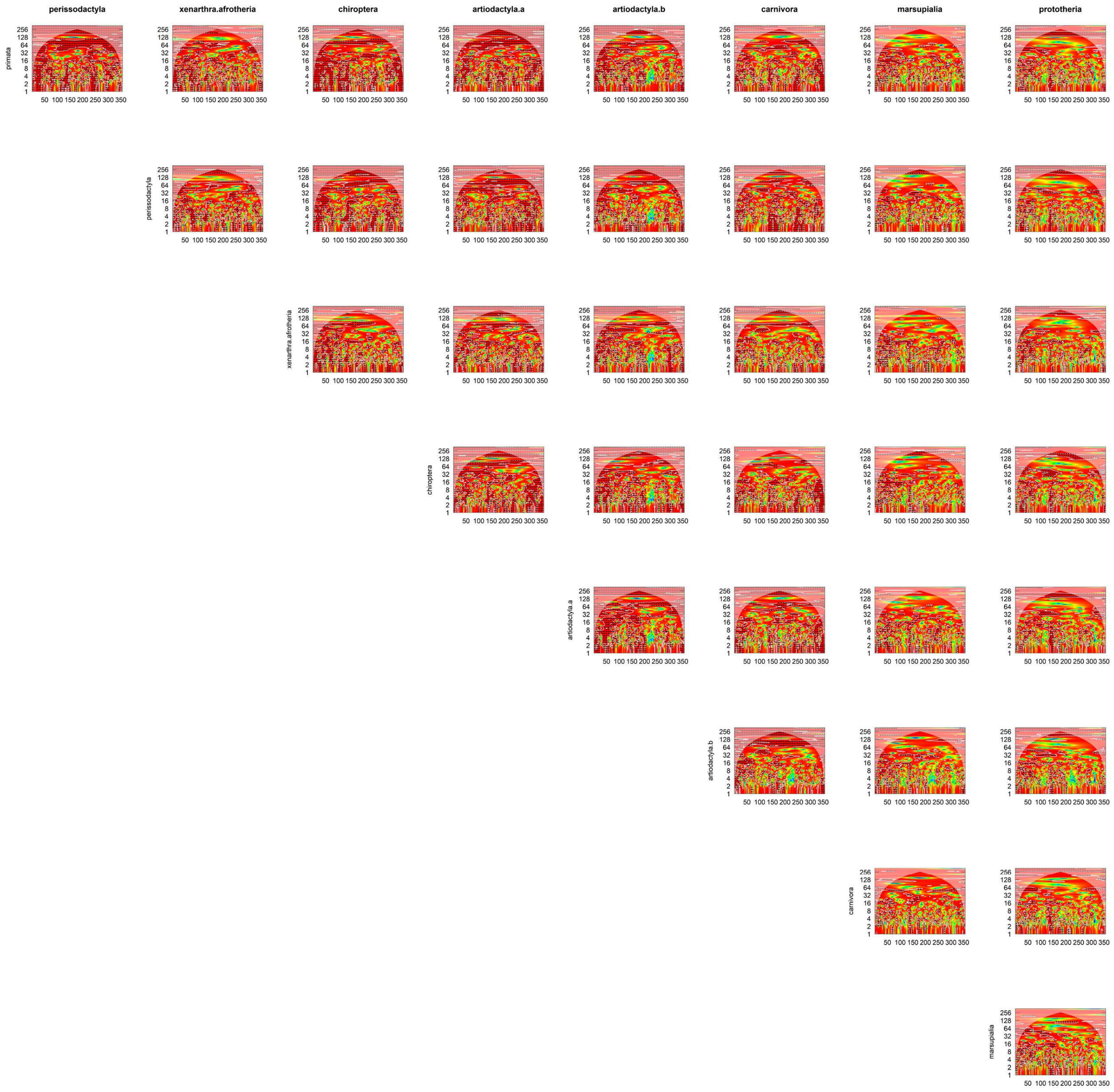
perissodactyla
xenarthra.afrotheria
chiroptera
artiodactyla.a
artiodactyla.b
carnivora
marsupialia
prototheria
primata
perissodactyla
xenarthra.afrotheria
chiroptera
artiodactyla.a
artiodactyla.b
carnivora
marsupialia
256
128
64
32
16
8
4
2
1
50 100 150 200 250 300 350

Figure S4D

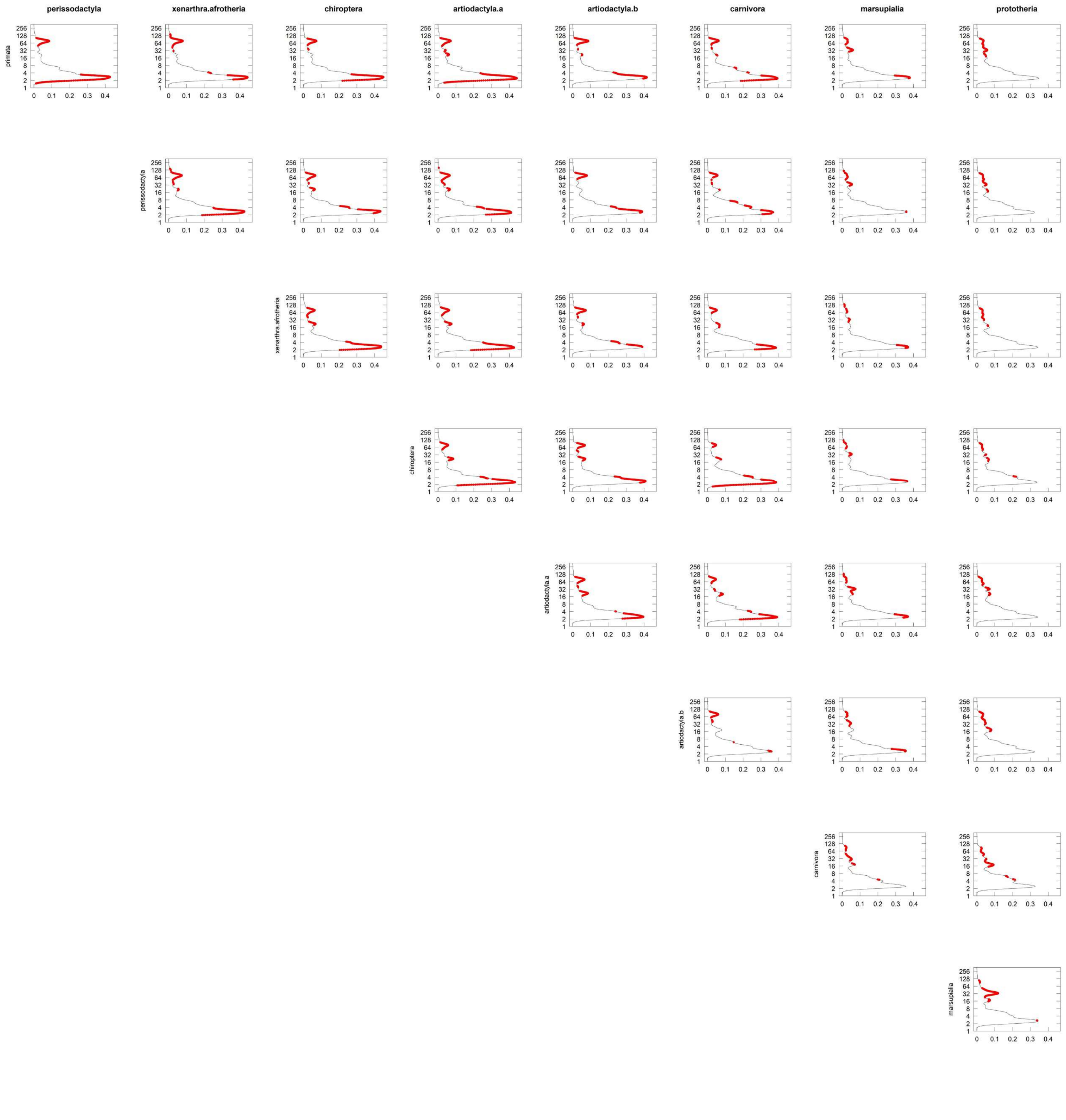
perissodactyla
xenarthra.afrotheria
chiroptera
artiodactyla.a
artiodactyla.b
carnivora
marsupialia
prototheria
primata
perissodactyla
xenarthra.afrotheria
chiroptera
artiodactyla.a
artiodactyla.b
carnivora
marsupialia

Figure S4E

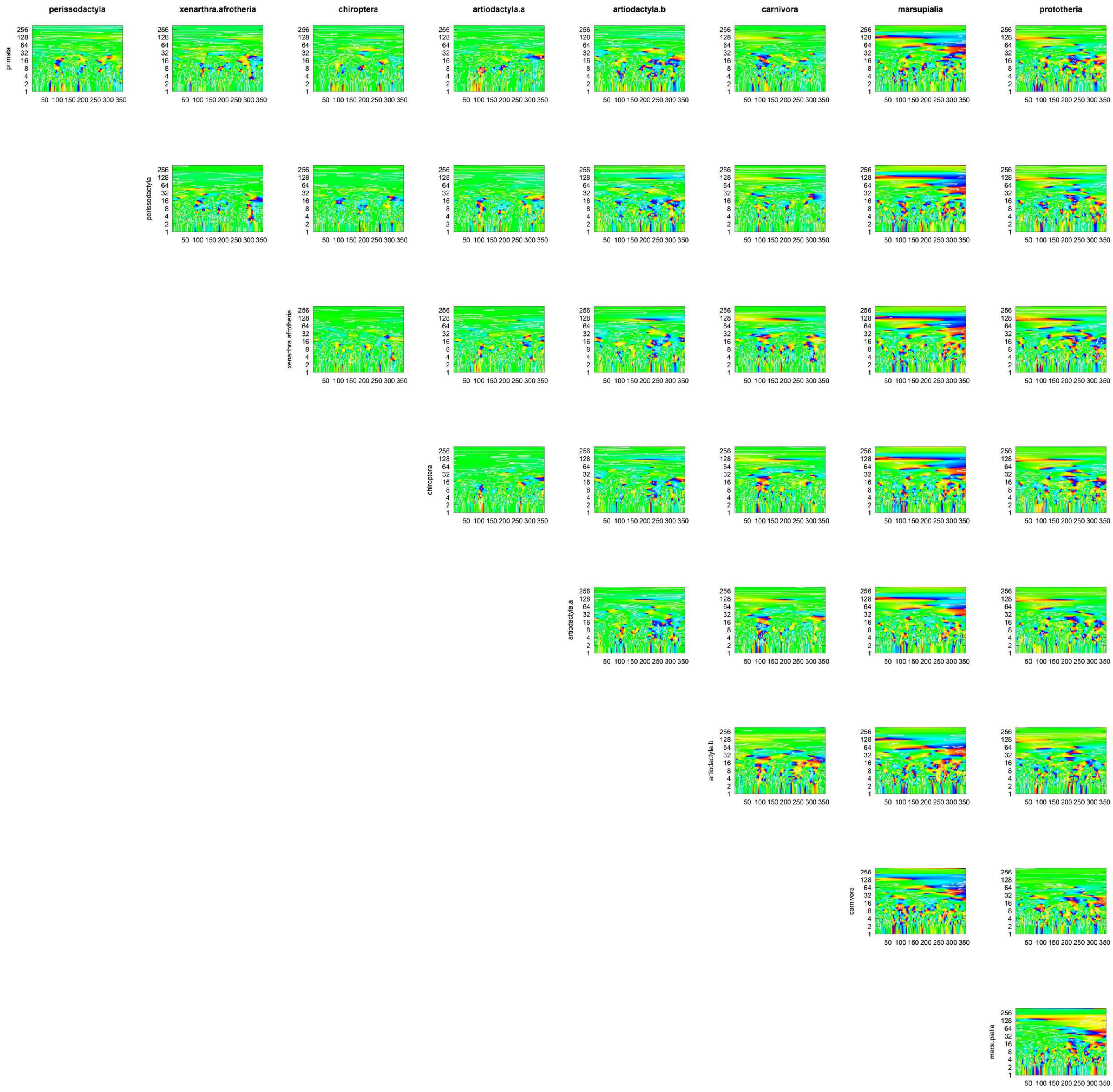
perissodactyla
xenarthra.afrotheria
chiroptera
artiodactyla.a
artiodactyla.b
carnivora
marsupialia
prototheria
primata
perissodactyla
xenarthra.afrotheria
chiroptera
artiodactyla.a
artiodactyla.b
carnivora
marsupialia

Figure S4F

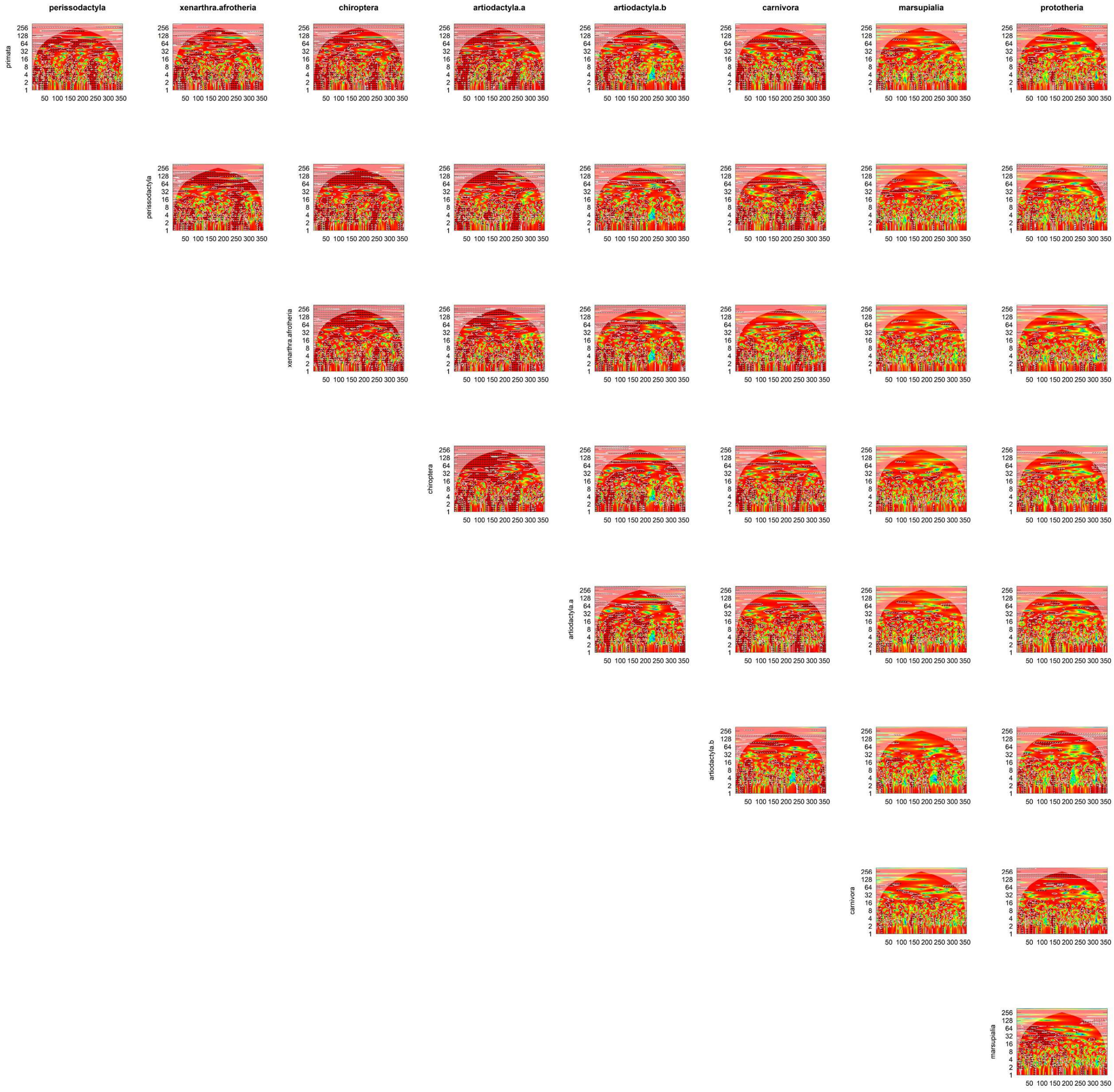

perissodactyla
xenarthra.afrotheria
chiroptera
artiodactyla.a
artiodactyla.b
carnivora
marsupialia
prototheria
primata
perissodactyla
xenarthra.afrotheria
chiroptera
artiodactyla.a
artiodactyla.b
carnivora
marsupialia

Figure S4G

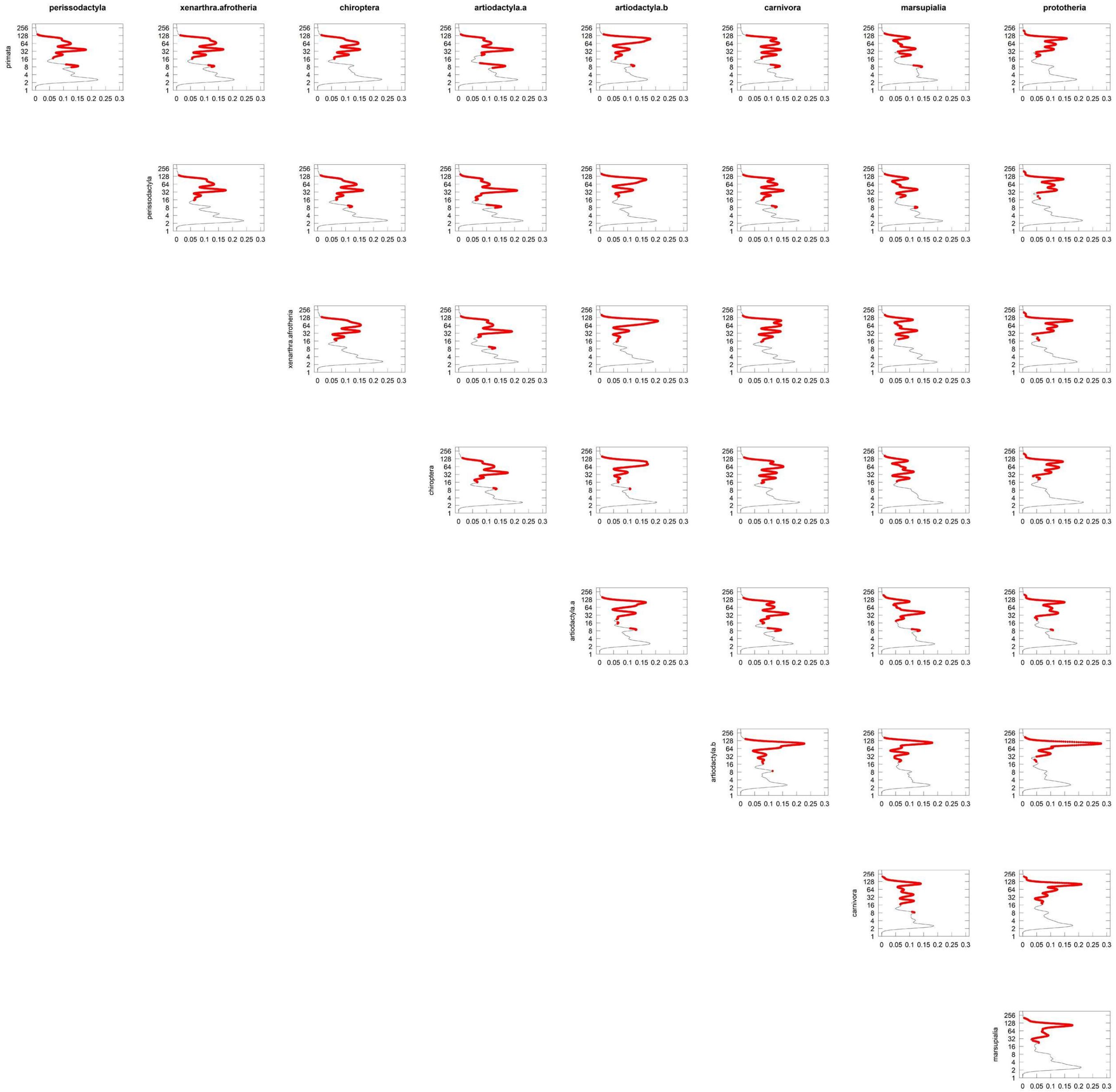
perissodactyla
xenarthra.afrotheria
chiroptera
artiodactyla.a
artiodactyla.b
carnivora
marsupialia
prototheria
primata
perissodactyla
xenarthra.afrotheria
chiroptera
artiodactyla.a
artiodactyla.b
carnivora
marsupialia
256
128
64
32
16
8
4
2
1
0 0.05 0.1 0.15 0.2 0.25 0.3

Figure S4H

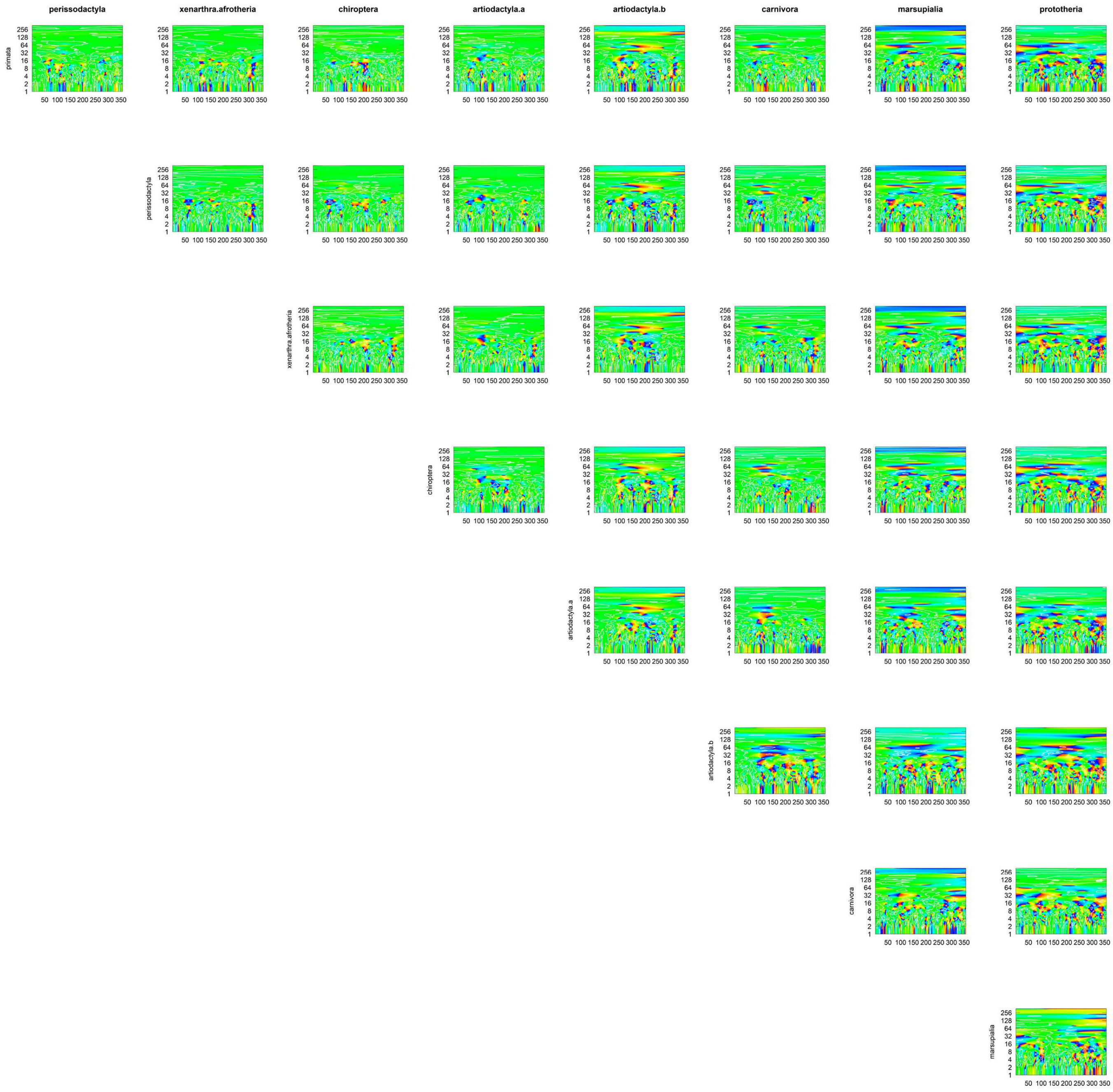

perissodactyla
xenarthra.afrotheria
chiroptera
artiodactyla.a
artiodactyla.b
carnivora
marsupialia
prototheria
primata
perissodactyla
xenarthra.afrotheria
chiroptera
artiodactyla.a
artiodactyla.b
carnivora
marsupialia
256 128 64 32 16 8 4 2 1
50 100 150 200 250 300 350

Figure S4I

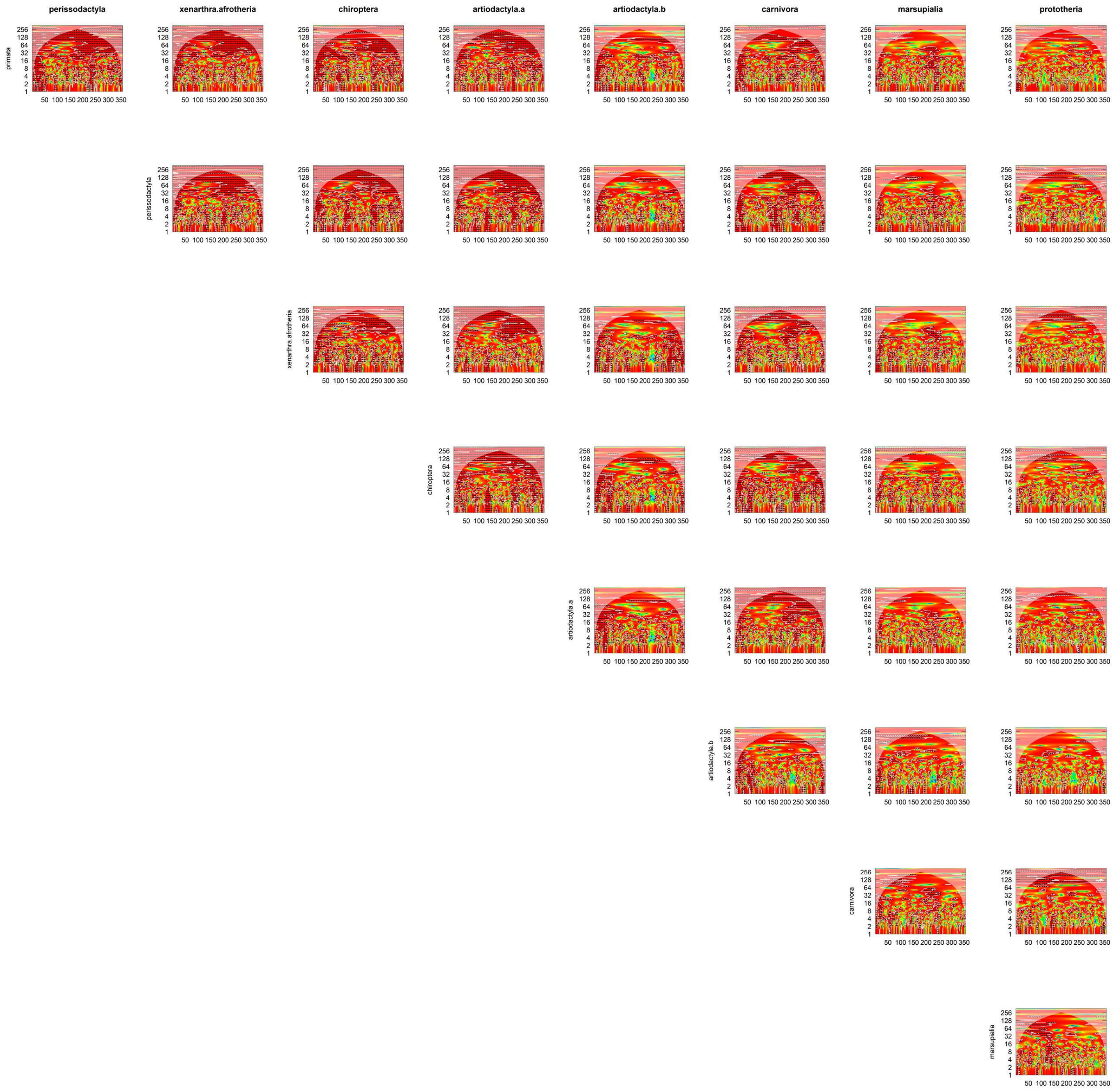
perissodactyla
xenarthra.afrotheria
chiroptera
artiodactyla.a
artiodactyla.b
carnivora
marsupialia
prototheria
primata
perissodactyla
xenarthra.afrotheria
chiroptera
artiodactyla.a
artiodactyla.b
carnivora
marsupialia

Figure S4J

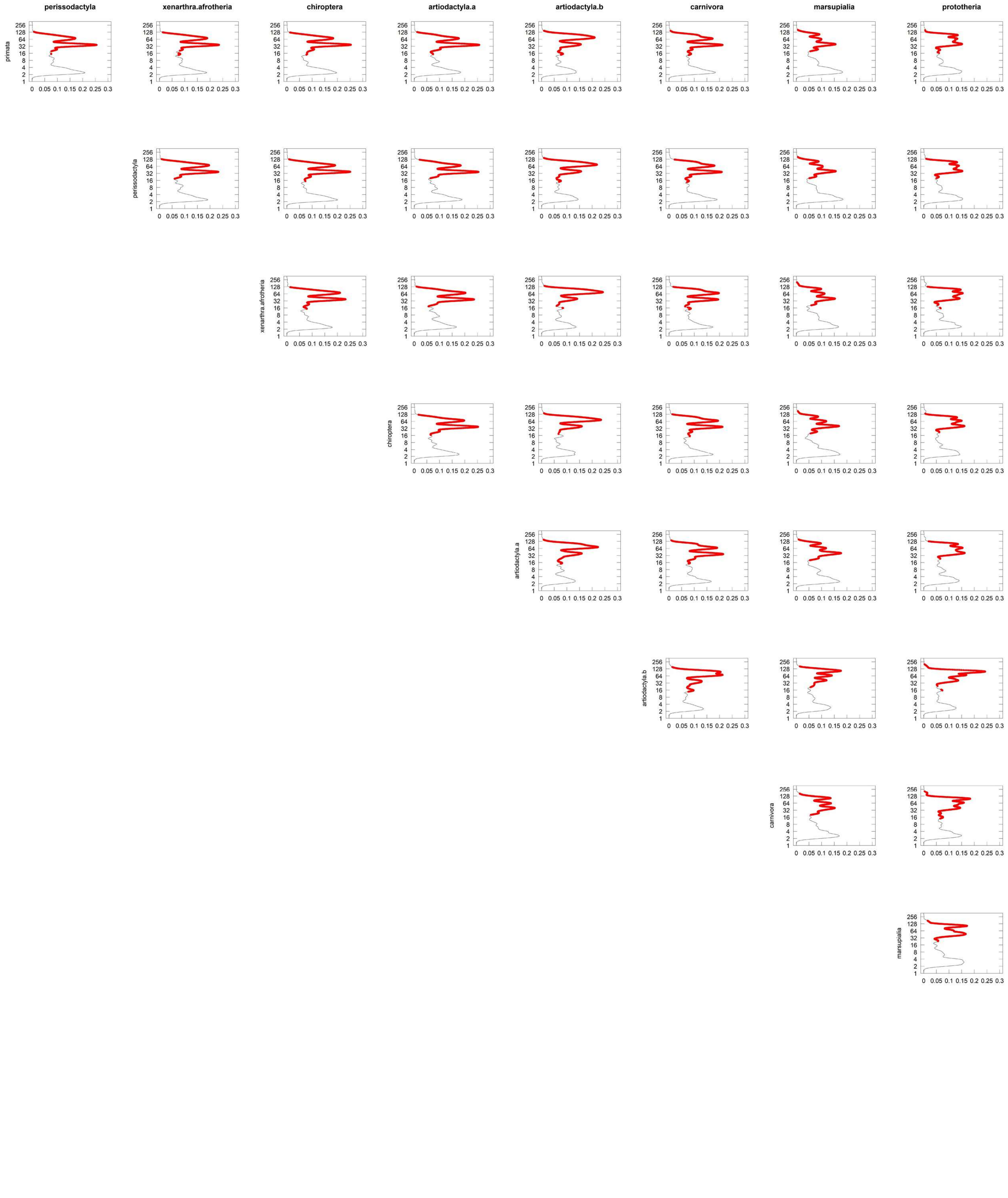
perissodactyla
xenarthra.afrotheria
chiroptera
artiodactyla.a
artiodactyla.b
carnivora
marsupialia
prototheria
primata
perissodactyla
xenarthra.afrotheria
chiroptera
artiodactyla.a
artiodactyla.b
carnivora
marsupialia

Figure S4K

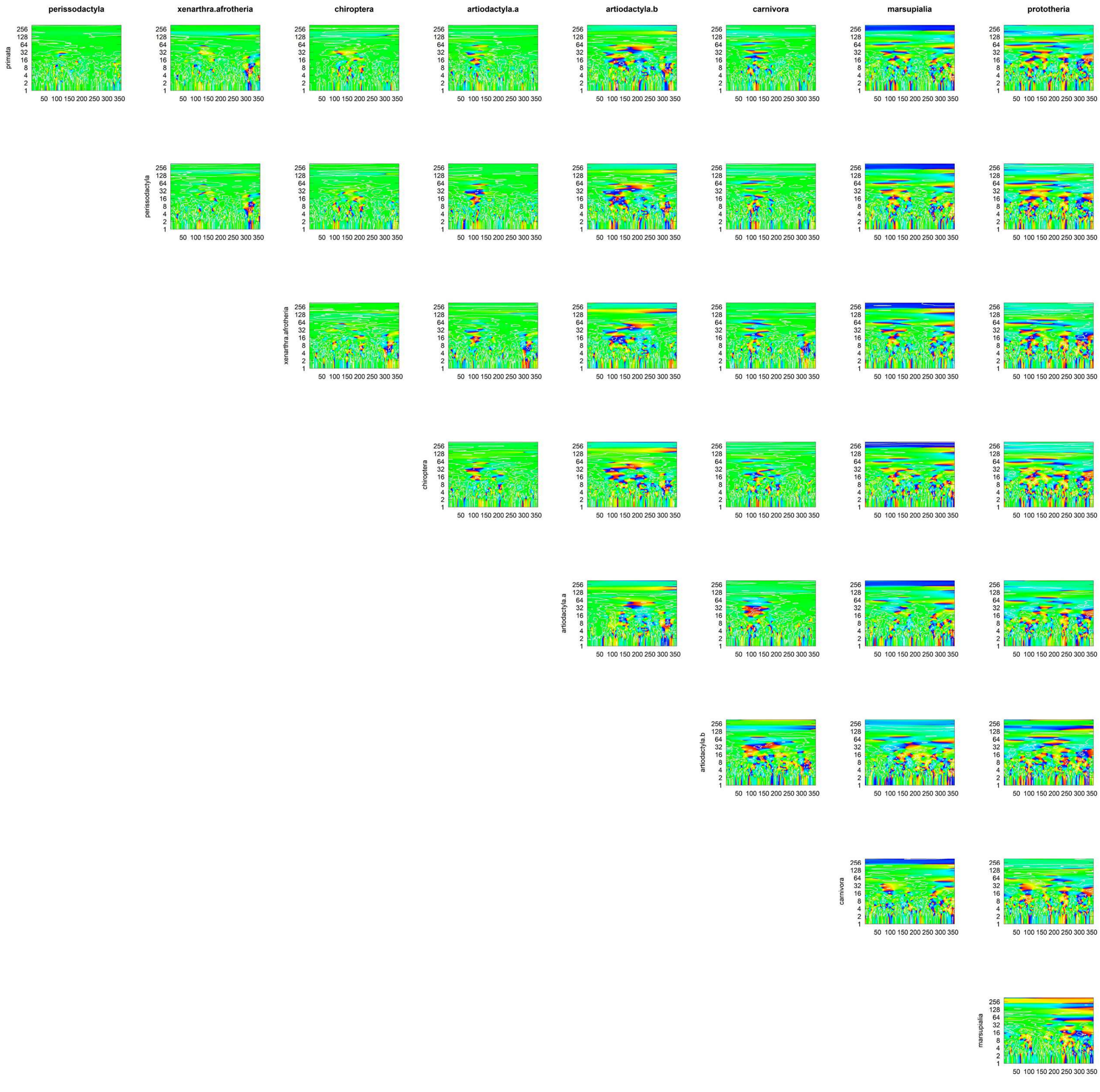

perissodactyla
xenarthra.afrotheria
chiroptera
artiodactyla.a
artiodactyla.b
carnivora
marsupialia
prototheria
primata
perissodactyla
xenarthra.afrotheria
chiroptera
artiodactyla.a
artiodactyla.b
carnivora
marsupialia
256 128 64 32 16 8 4 2 1
50 100 150 200 250 300 350

Figure S4L

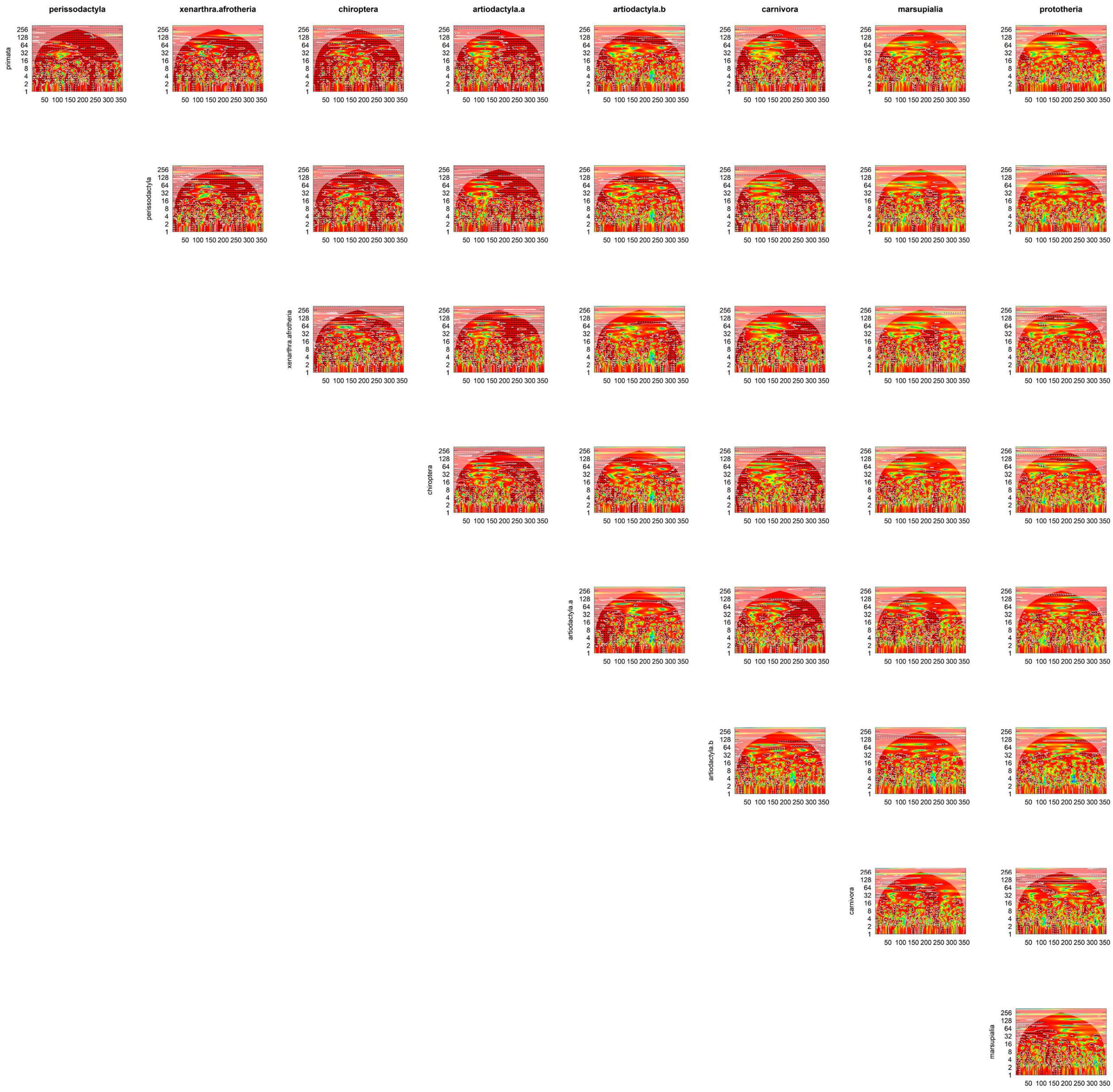

perissodactyla
xenarthra.afrotheria
chiroptera
artiodactyla.a
artiodactyla.b
carnivora
marsupialia
prototheria
primata
perissodactyla
xenarthra.afrotheria
chiroptera
artiodactyla.a
artiodactyla.b
carnivora
marsupialia

Figure S5

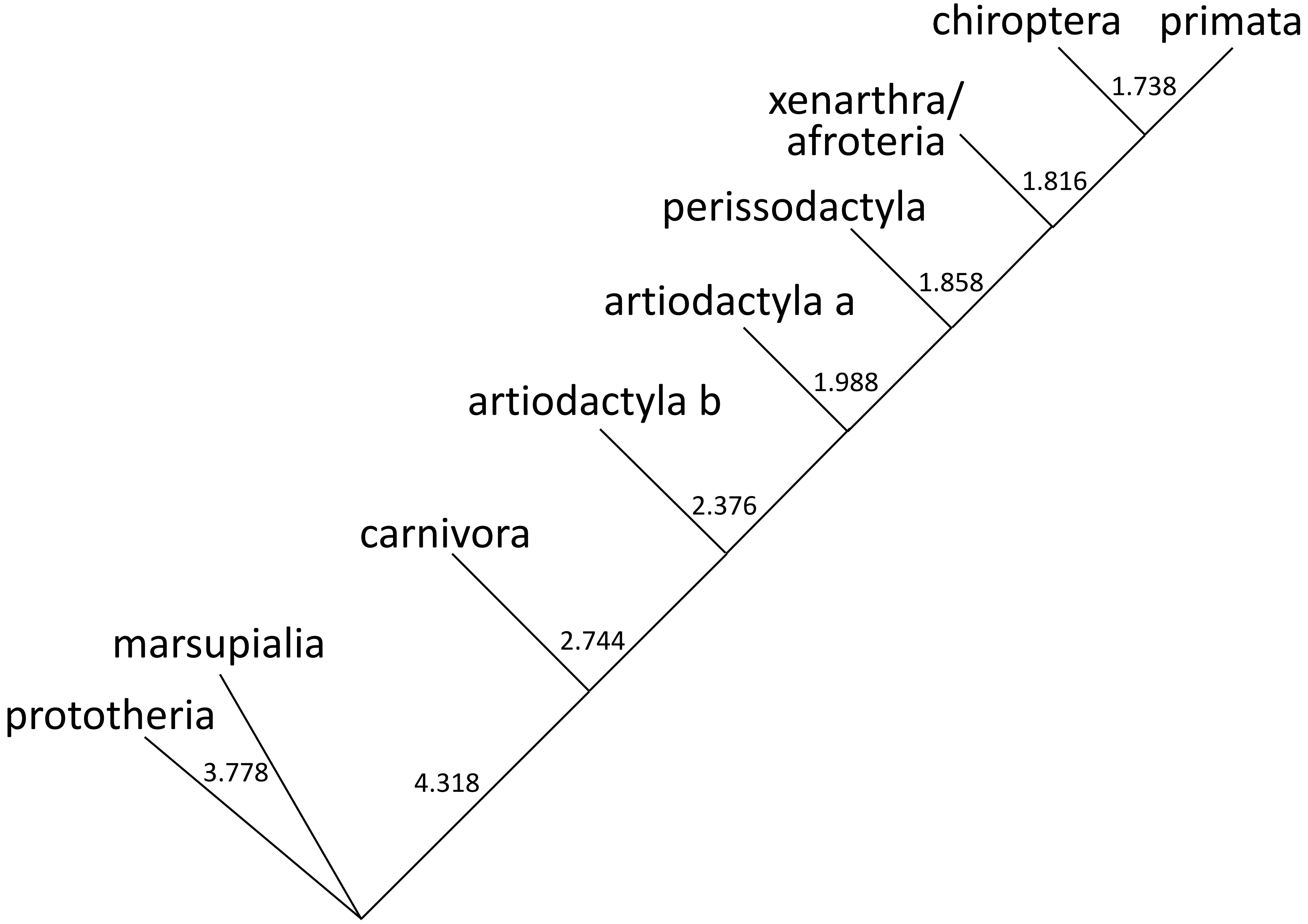
chiroptera
primata
1.738
xenarthra/
afroteria
1.816
perissodactyla
1.858
artiodactyla a
1.988
artiodactyla b
2.376
carnivora
2.744
marsupialia
prototheria
3.778
4.318

Figure S6A

```
CLUSTAL O(1.2.4) multiple sequence alignment


Aves_1          MKLALLCLCFISIAAAWPVSKSKQHAISASSEEKYDPRSHHAHRYHQDHVDSQSQEHLQQ    60
Aves_3          MKVAVLCLCLISITAAWPVIQSKQHAISASSEEKYDSRGHHLHRYHNDHVNSQSQESQQH    60
Aves_2          MKVAVLCLCLISITVAWPVSKSKQHAVSASSEEKYDSRGHHSHRH-HHHVNSQSLESLLL    59
Aves_4          MKVAVLCLCLISITAAWPVSKSKQHAISASSEEKYDPRGHHSHRYHHHHVNSQSWESLQH    60
                **:*:****:***:.**** :*****:********* *.** **: :.**:*** *

Aves_1          TQNDLASLQQTHYSSEENADVPEQPDFPDIPSKSQEAVDDDD-DDDNDSNDTDESDEVVT    119
Aves_3          PQSDLASSQQTLYSSEESVDVPEQLHFPDVSSKSHEDVDDDDDDDDNDSNDTDESEEVVT    120
Aves_2          PRDDLASPQQTLYSSEERAVVLVPERFPAVFSKSHEDPDDD--VDDNDSNDTDESDEVVT    117
Aves_4          PQNDLASPQQTLYSSEESVDVPVQPHFPDVSSKSHEDVDDD--DDDNDSNDTDESDEVVT    118
                 :.**** *** ***** . *     ** : ***:*  ***   ***********:****

Aves_1          DFPTEAPV------TPFNRGDNAGRGDSVAYGFRAKAHVVKASKLRKAARKLIEDDATAE    173
Aves_3          SFPTDIPVTEPFPTFPFTRGDNAGRGDSVAYRMRAKAALLKSIKLHKAAKKL-IYDATEE    179
Aves_2          VFPTDAPVTAPFPPYPFTRGDNAGRGDSVAYRIKAKAAVVKPSKLHKAARKLIVHDATEE    177
Aves_4          SFPTDIPVTVPFPPFPFTRGDNAGRGDSVAYRVRAKATVVKSSKLRKAAKKLIVYDATEE    178
                 ***: **       **.************* .:*** ::*  **:***:**   *** *

Aves_1          VG-----D-------------------SQLAGLWWPKESREQDSREL---AQHQSVENDS    206
Aves_3          DESDMDADSQ-RSVSREDSASRSSLRKHASSV-VWSEQSHGRDSSEQDSDPHHRSLENDS    237
Aves_2          DESALAADSHQAGLSREDLGAHRSAGKYADSG-EWGDKSHGHDSNEVVRKLPDRSLENDS    236
Aves_4          DESALDADSQQAGLSQEDPAARRSLGKHAISG-EWADKSHGQDSSELDSKQRDRSMENDS    237
                       *                      :   * .:*: :** *      .:*:****

Aves_1          RPRFDSPEVDGGDSKASAGVDS------------------------------RESQ----    232
Aves_3          RHKSDSHEAEGDSSKSGVRGDSLSSVESKERGDSHPSVESRESRDSHPSVESRESRDRVS    297
Aves_2          RQKFDSPEVEVGSSERGARGDSP------------------------PSRESRESQARVS    272
Aves_4          RQKFDSHEVEGDDSKAGVRGDSH------------------------QSMESRESQVRVS    273
                * : ** *.: ..*: ..  **                              ***:

Aves_1          GSVPAVDTSNQTLESAEDAEDRHSIENNEVTR       264
Aves_3          AELSD-DISNQTLESAEDSQDRHSIESNEVTL       328
Aves_2          AEVPDGDNSNQTLESAEDAQDHLSIKHNEVIF       304
Aves_4          AEIPD-DNSNQTLESAEDAQDHHSIENNEVTL       304
                ..:   * **********::*: **: ***
```

Figure S6B

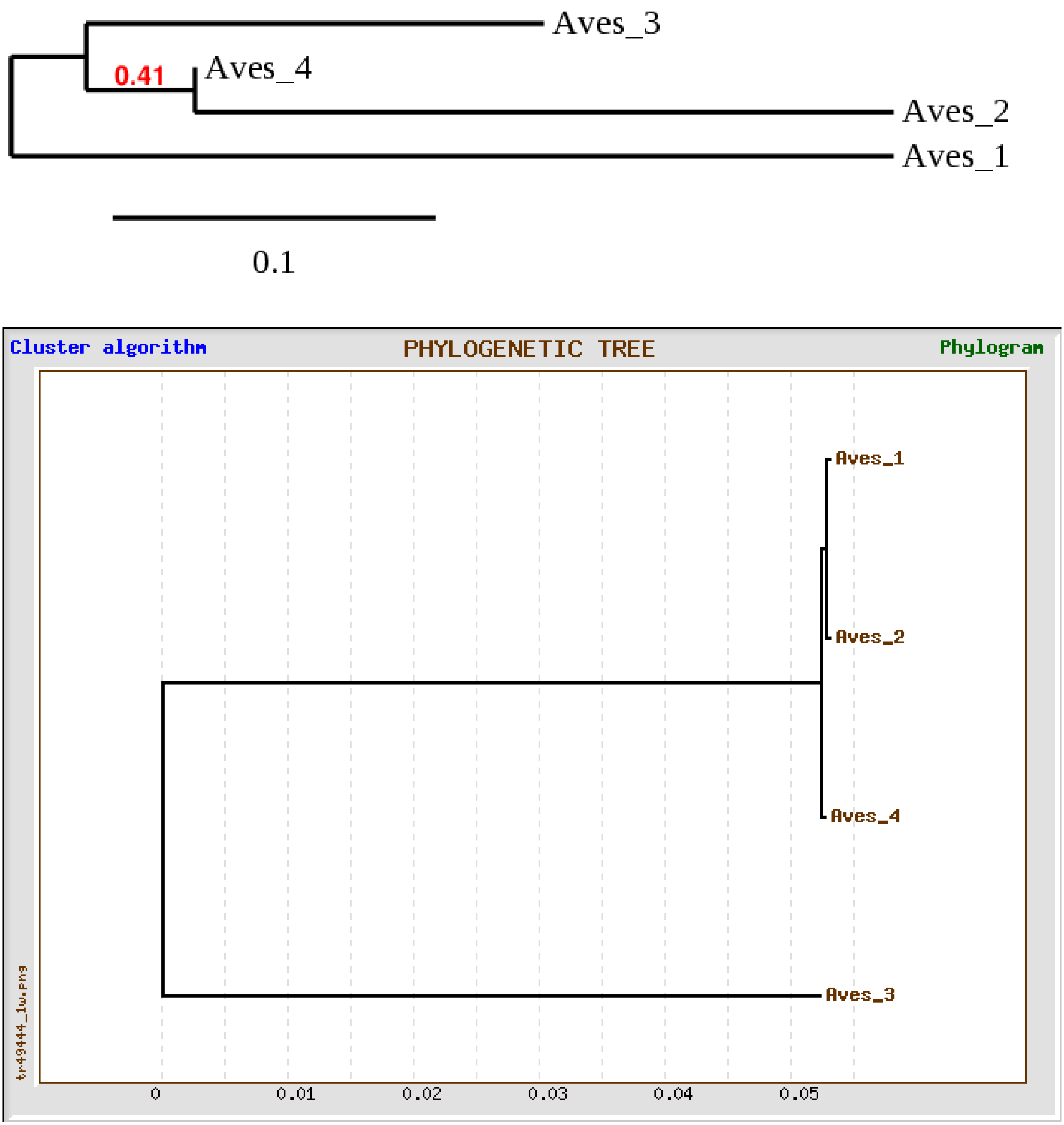
Aves_3
0.41
Aves_4
Aves_2
Aves_1
0.1
Cluster algorithm
PHYLOGENETIC TREE
Phylogram
Aves_1
Aves_2
Aves_4
Aves_3
0
0.01
0.02
0.03
0.04
0.05

Figure S6C

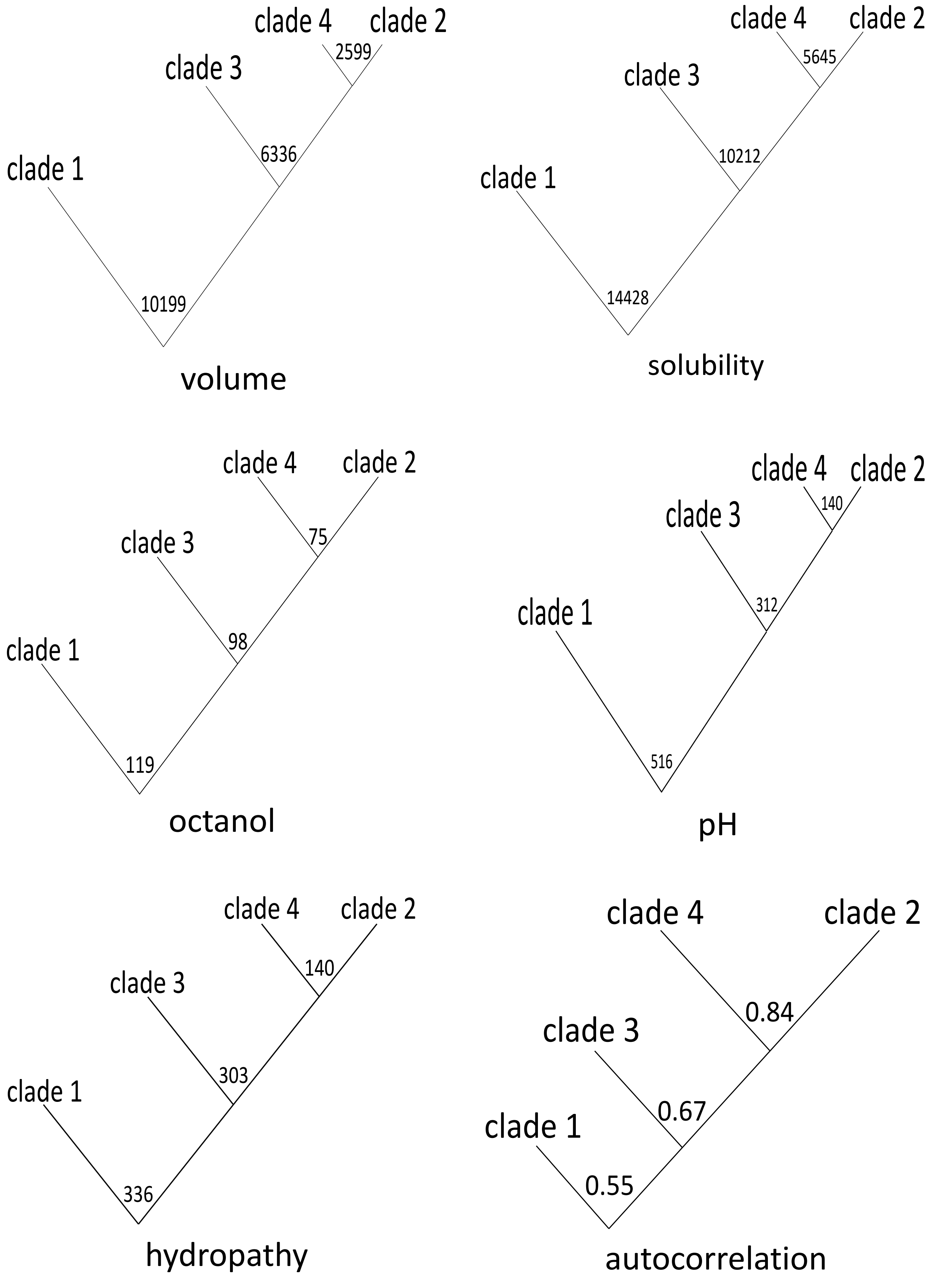
clade 4
clade 2
2599
clade 3
6336
clade 1
10199
volume
clade 4
clade 2
5645
clade 3
10212
clade 1
14428
solubility
clade 4
clade 2
75
clade 3
98
clade 1
119
octanol
clade 4
clade 2
140
clade 3
312
clade 1
516
pH
clade 4
clade 2
140
clade 3
303
clade 1
336
hydropathy
clade 4
clade 2
0.84
clade 3
0.67
clade 1
0.55
autocorrelation

Figure S6D

hydropathy

	clade 1	clade 2	clade 3	clade 4
clade 1	1	0.716317	0.7272	0.812583
clade 2		1	0.65021	0.793211
clade 3			1	0.7731
clade 4				1

octanol

	clade 1	clade 2	clade 3	clade 4
clade 1	1	0.635604	0.689936	0.782999
clade 2		1	0.69394	0.809304
clade 3			1	0.802931
clade 4				1

pH

	clade 1	clade 2	clade 3	clade 4
clade 1	1	0.440175	0.286539	0.504615
clade 2		1	0.457432	0.861393
clade 3			1	0.734527
clade 4				1

solubility

	clade 1	clade 2	clade 3	clade 4
clade 1	1	0.648303	0.525981	0.694438
clade 2		1	0.708517	0.856875
clade 3			1	0.712471
clade 4				1

volume

	clade 1	clade 2	clade 3	clade 4
clade 1	1	0.474397	0.200967	0.479151
clade 2		1	0.360802	0.898514
clade 3			1	0.76309
clade 4				1

average

	clade 1	clade 2	clade 3	clade 4
clade 1	1	0.582959	0.486125	0.654757
clade 2		1	0.57418	0.843859
clade 3			1	0.757224
clade 4				1

Figure S7A

```
CLUSTAL O(1.2.4) MULTIPLE SEQUENCE ALIGNMENT

PARAPOXVIRUS_DQ888328.1            MRLLI---QCSVVLILCLIQIHWIYASSGGSS--NSGGNLADWMQTSDKSKCMPRDTVVY    55
STOMATITISVIRUS_AY513237.1         MKCLIVCMQWSLALLLCLQCVKWMQAAPATSNND--NSRVTPWTEVYGNSHCRPMPTTVR    58
TRIMERESURUS_AB154419.1            MNFLLTWIHWGLAALLYFHNAKVLQAAPAQGDGDRQQSEVIPFMTVYERSVCRPIETMVD    60
SPALAX_AF186236.1                  MNFLLSWMHWTLALLLYLHHAKWSQAAPTAEGE-QKPHEVVKFMDVFRRSYCHPIETLVD    59
HOMO_ AY047581.1                   MNFLLSWVHWSLALLLYLHHAKWSQAAPMAEGGGQNHHEVVKFMDVYQRSYCHPIETLVD    60
PILIOCOLOBUS_XP_023068672.1        MNFLLSWVHWSLALLLYLHHAKWSQAAPMAEGGGQNRHEVVKFMDVYQRSYCHPIETLVD    60
SUS_JF831364.1                     MNFLLSWVHWSLALLLYLHHAKWSQAAPMAEGD-QKPHEVVKFMDVYQRSYCRPIETLVD    59
GALLUS_ AB011078.1                 MNFLLTWIHWGLAALLYLQSAELSKAAPALGDGERKPNEVIKFLEVYERSFCRTIETLVD    60
PASSER_KY001973.1                  MNFLLTWIRWGLAALLYLQSAELSKAAPALGDGERKPNEVIKFLEVYERSFCRTIETLVD    60
XENOPUS_AF008594.1                 MNFLPSWIHWGLAVLLYIPHAQLSGAAPMPGEGDHKPTEVVKFLKVYERSMCQVREILVD    60
                                      :   ::  :. :* :   .   *:           .:  :  .  .* *      *

PARAPOXVIRUS_DQ888328.1            IGNEYPGSVGERYNPQCVTVKKCGGCCNGDNKVCTSTETANTTVTVMVTGVSSS------    109
STOMATITISVIRUS_AY513237.1         VSDEYPNDTSERYNPQCVTLMRCGGCCNDESLECVPTETSNVTMQLMVTSAHNGGSNDNG    118
TRIMERESURUS_AB154419.1            IFQDYPDEVEYILKPPCVALMRCGGCCNDEALECVPTELYNVTMEIMKLKPYQS------    114
SPALAX_AF186236.1                  IFQEYPDEIEYIFKPSCVPLMRCGGCCNDEALECVPTSESNITMQIMRIKPHQS------    113
HOMO_ AY047581.1                   IFQEYPDEIEYIFKPSCVPLMRCGGCCNDEGLECVPTEESNITMQIMRIKPHQG------    114
PILIOCOLOBUS_XP_023068672.1        IFQEYPDEIEYIFKPSCVPLMRCGGCCNDEGLECVPTEESNITMQIMRIKPHQG------    114
SUS_JF831364.1                     IFQEYPDEIEYIFKPSCVPLMRCGGCCNDEGLECVPTEEFNITMQIMRIKPHQG------    113
GALLUS_ AB011078.1                 IFQEYPDEVEYIFRPSCVPLMRCAGCCGDEGLECVPVDVYNVTMEIARIKPHQS------    114
PASSER_KY001973.1                  IFQEYPDEVEYIFKPSCVPLMGCAGCCGDEGLECVPVDVYNVTMEIMRIKPHQS------    114
XENOPUS_AF008594.1                 IFQEYPDEVEYIFKPSCVPLMRCAGCCNDESLECVPTECYNITMQIMKIKPHIS------    114
                                   : ::**..     .* ** :  *.***..:   *. ..  * *: :       .

PARAPOXVIRUS_DQ888328.1            SGNGASSNFRDISISEDKKCECREKFTTTPPTTTMPPRR---------------------    148
STOMATITISVIRUS_AY513237.1         SGGGIGSGMREMSFLQHNKCECKPKSTPPPETTEESHRR---------------------    157
TRIMERESURUS_AB154419.1            ------QHIHPMSFQQHSKCECRPKKETRIIQEKCEKPRR--------------------    148
SPALAX_AF186236.1                  ------QHIGEMSFLQHNRCECRPKKDRTRL-------------------------ENHC    142
HOMO_ AY047581.1                   ------QHIGEMSFLQHNKCECRPKKDRARQ-------------------------ENPC    143
PILIOCOLOBUS_XP_023068672.1        ------QHIGEMSFLQHNKCECRPKKDRARQ-------------------------ENPC    143
SUS_JF831364.1                     ------QHIGEMSFLQHNKCECRPKKDRARQ-------------------------ENPC    142
GALLUS_ AB011078.1                 ------QHIAHMSFLQHSKCDCRPKKDVKNKQEKKSKRGKGKGQKRKRKKGRYKPPSFHC    168
PASSER_KY001973.1                  ------QHISHMSFLQHSKCDCRPKKDVKNKQEKKSKRGRGKGQKRKRKKGRYKPLSFHC    168
XENOPUS_AF008594.1                 ------QHIMDMSFQQHSQCECRPKKEVKSKQE------------------------NHC    144
                                         . :  :*: :..:*:*: *

PARAPOXVIRUS_DQ888328.1            --------------------------------------------------      148
STOMATITISVIRUS_AY513237.1         --------------------------------------------------      157
TRIMERESURUS_AB154419.1            --------------------------------------------------      148
SPALAX_AF186236.1                  EPCSE--RRKHLFVQDPQTCKCSCKNTDSRCKARQLELNERTCRCDKPRR      190
HOMO_ AY047581.1                   GPCSE--RRKHLFVQDPQTCKCSCKNTDSRCKARQLELNERTCRCDKPRR      191
PILIOCOLOBUS_XP_023068672.1        GPCSE--RRKHLFVQDPQTCKCSCKNTDSRCKARQLELNERTCRCDKPRR      191
SUS_JF831364.1                     GPCSE--RRKHLFVQDPQTCKCSCKNTDSRCKARQLELNERTCRCDKPRR      190
GALLUS_ AB011078.1                 EPCSE--RRKHLFVQDPQTCKCSCKFTDSRCKSRQLELNERTCRCEKPRR      216
PASSER_KY001973.1                  EPCSE--RRKHLFVQDPQTCKCSCKFTDSRCKSRQLELNERTCRCEKPRR      216
XENOPUS_AF008594.1                 EPCTEKSQRKHLFVQDPQTCKCSCKNTDSRCKTRQLELNERTCRCEKPRR      194
```

Figure S7B

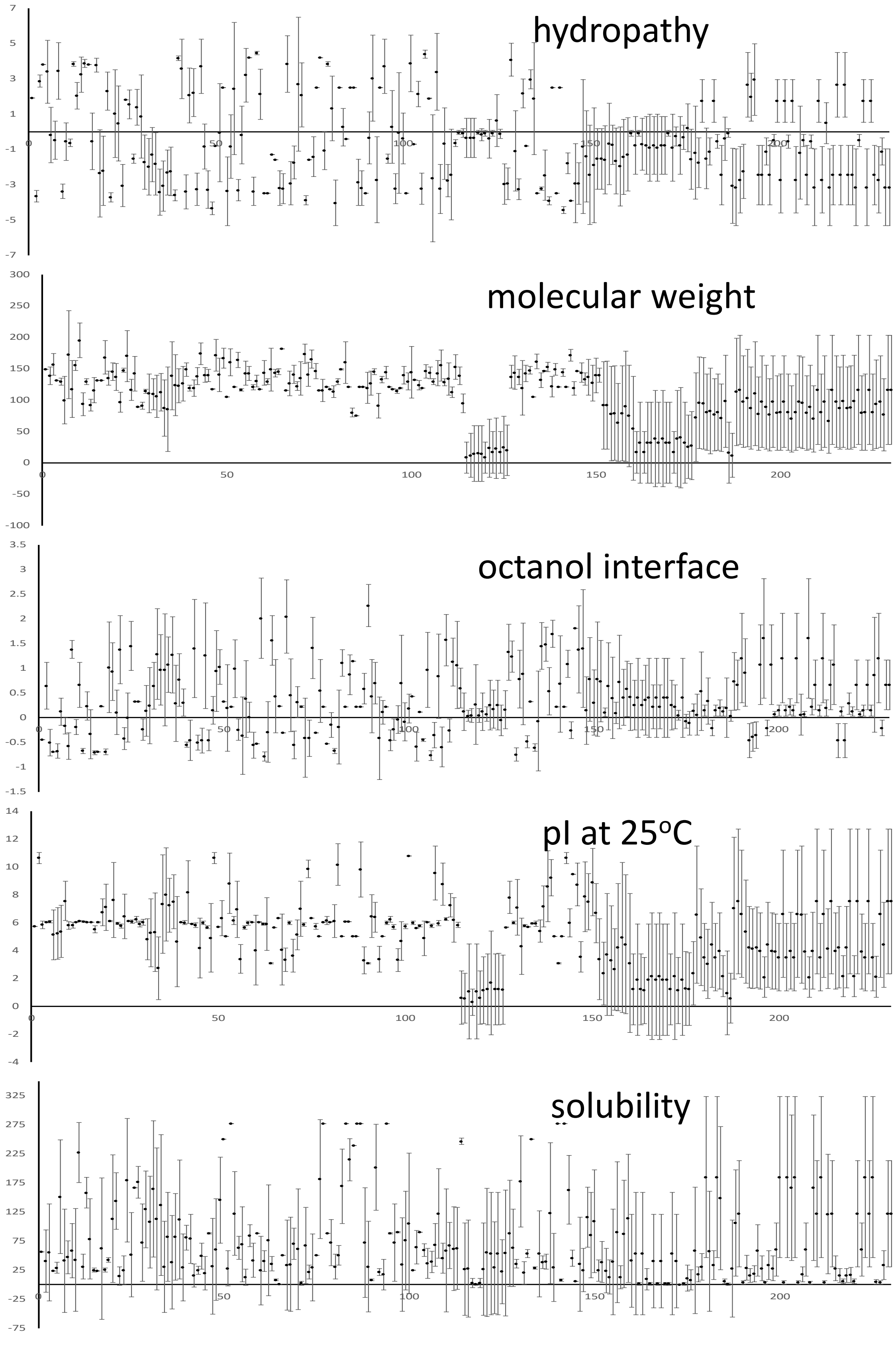
hydropathy
molecular weight
octanol interface
pI at 25oC
solubility

Figure S7C

hydropathy

	homo	pilocolobus	sus	spalax	xenopus	passer	gallus	timeresurus	stomatitis virus	parapoxvirus
homo	1.00	1.00	1.00	0.97	0.84	0.88	0.76	0.58	0.47	0.37
pilocolobus		1.00	1.00	0.97	0.84	0.75	0.76	0.58	0.47	0.56
sus			1.00	0.97	0.83	0.75	0.76	0.57	0.70	0.56
spalax				1.00	0.82	0.74	0.75	0.59	0.44	0.54
xenopus					1.00	0.76	0.77	0.75	0.57	0.53
passer						1.00	0.99	0.56	0.40	0.51
gallus							1.00	0.57	0.41	0.29
timeresurus								1.00	0.69	0.55
stomatitis v									1.00	0.65
parapoxvirus										1.00

octanol

	homo	pilocolobus	sus	spalax	xenopus	passer	gallus	timeresurus	stomatitis virus	parapoxvirus
homo	1.00	1.00	0.99	0.93	0.79	0.76	0.64	0.51	0.34	0.27
pilocolobus		1.00	0.99	0.93	0.80	0.65	0.64	0.51	0.35	0.39
sus			1.00	0.97	0.78	0.67	0.66	0.48	0.52	0.38
spalax				1.00	0.79	0.64	0.64	0.50	0.31	0.37
xenopus					1.00	0.65	0.64	0.62	0.45	0.36
passer						1.00	1.00	0.46	0.23	0.23
gallus							1.00	0.46	0.23	0.13
timeresurus								1.00	0.60	0.33
stomatitis v									1.00	0.41
parapoxvirus										1.00

pI

	homo	pilocolobus	sus	spalax	xenopus	passer	gallus	timeresurus	stomatitis virus	parapoxvirus
homo	1.00	1.00	1.00	0.99	0.86	0.42	0.58	0.37	0.15	0.22
pilocolobus		1.00	0.99	0.98	0.85	0.57	0.58	0.37	0.16	0.18
sus			1.00	0.98	0.84	0.58	0.60	0.37	0.13	0.19
spalax				1.00	0.83	0.57	0.59	0.36	0.15	0.19
xenopus					1.00	0.49	0.50	0.30	0.07	0.17
passer						1.00	0.99	0.36	-0.16	0.03
gallus							1.00	0.37	-0.13	0.08
timeresurus								1.00	0.71	0.77
stomatitis v									1.00	0.77
parapoxvirus										1.00

solubility

	homo	pilocolobus	sus	spalax	xenopus	passer	gallus	timeresurus	stomatitis virus	parapoxvirus
homo	1.00	1.00	0.94	0.90	0.80	0.74	0.70	0.37	0.22	0.22
pilocolobus		1.00	0.93	0.89	0.79	0.69	0.70	0.37	0.22	0.22
sus			1.00	0.93	0.82	0.70	0.71	0.38	0.22	0.21
spalax				1.00	0.80	0.66	0.67	0.34	0.23	0.20
xenopus					1.00	0.70	0.71	0.61	0.22	0.18
passer						1.00	0.98	0.40	0.21	0.13
gallus							1.00	0.39	0.21	0.13
timeresurus								1.00	0.43	0.40
stomatitis v									1.00	0.58
parapoxvirus										1.00

MW

	homo	pilocolobus	sus	spalax	xenopus	passer	gallus	timeresurus	stomatitis virus	parapoxvirus
homo	1.00	1.00	1.00	0.99	0.84	0.40	0.76	0.46	0.26	0.33
pilocolobus		1.00	1.00	0.99	0.84	0.74	0.76	0.46	0.26	0.25
sus			1.00	0.99	0.83	0.74	0.76	0.10	0.20	0.26
spalax				1.00	0.83	0.73	0.76	0.44	0.24	0.24
xenopus					1.00	0.69	0.71	0.32	0.17	0.22
passer						1.00	0.98	0.53	0.00	0.06
gallus							1.00	0.54	0.02	0.16
timeresurus								1.00	0.90	0.89
stomatitis v									1.00	0.86
parapoxvirus										1.00

average

	homo	pilocolobus	sus	spalax	xenopus	passer	gallus	timeresurus	stomatitis virus	parapoxvirus
homo	1	1.00	0.98	0.96	0.83	0.64	0.69	0.46	0.29	0.28
pilocolobus		1	0.98	0.95	0.82	0.68	0.69	0.46	0.29	0.32
sus			1	0.97	0.82	0.69	0.70	0.38	0.35	0.32
spalax				1	0.81	0.67	0.68	0.45	0.28	0.31
xenopus					1	0.66	0.67	0.52	0.30	0.29
passer						1	0.99	0.46	0.14	0.19
gallus							1	0.47	0.15	0.16
timeresurus								1	0.67	0.59
stomatitis v									1	0.66
parapoxvirus										1

Figure S7D

hydropathy

	homo	pilocolobus	sus	spalax	xenopus	passer	gallus	timeresurus	stomatitis virus	parapoxvirus
homo	1.000	1.819	1.768	1.636	1.244	1.120	1.148	0.725	0.505	0.305
pilocolobus		1.000	1.759	1.628	1.229	1.111	1.139	0.728	0.505	0.307
sus			1.000	1.630	1.241	1.116	1.143	0.708	0.499	0.292
spalax				1.000	1.218	1.065	1.093	0.694	0.478	0.309
xenopus					1.000	1.166	1.221	0.706	0.409	0.265
passer						1.000	1.722	0.689	0.456	0.224
gallus							1.000	0.714	0.450	0.228
timeresurus								1.000	0.784	0.643
stomatitis v									1.000	0.665
parapoxvirus										1.000

octanol

	homo	pilocolobus	sus	spalax	xenopus	passer	gallus	timeresurus	stomatitis virus	parapoxvirus
homo	1.000	1.831	1.741	1.539	1.134	0.975	0.958	0.662	0.427	0.276
pilocolobus		1.000	1.741	1.538	1.134	0.975	0.958	0.653	0.427	0.280
sus			1.000	1.544	1.125	1.001	0.986	0.632	0.422	0.267
spalax				1.000	1.131	0.988	0.965	0.641	0.411	0.274
xenopus					1.000	0.996	0.987	0.664	0.334	0.271
passer						1.000	1.848	0.739	0.318	0.218
gallus							1.000	0.730	0.327	0.224
timeresurus								1.000	0.538	0.432
stomatitis v									1.000	0.518
parapoxvirus										1.000

pI

	homo	pilocolobus	sus	spalax	xenopus	passer	gallus	timeresurus	stomatitis virus	parapoxvirus
homo	1.000	1.738	1.663	1.522	1.102	0.882	0.894	0.542	0.353	0.197
pilocolobus		1.000	1.659	1.518	1.098	0.888	0.898	0.540	0.355	0.193
sus			1.000	1.573	1.107	0.907	0.920	0.531	0.371	0.200
spalax				1.000	1.128	0.903	0.917	0.521	0.335	0.216
xenopus					1.000	1.000	0.998	0.601	0.310	0.241
passer						1.000	1.602	0.578	0.311	0.152
gallus							1.000	0.576	0.325	0.178
timeresurus								1.000	0.670	0.609
stomatitis v									1.000	0.663
parapoxvirus										1.000

solubility

	homo	pilocolobus	sus	spalax	xenopus	passer	gallus	timeresurus	stomatitis virus	parapoxvirus
homo	1.000	1.426	1.357	1.199	0.830	0.746	0.749	0.368	0.256	0.153
pilocolobus		1.000	1.339	1.182	0.822	0.730	0.733	0.361	0.259	0.154
sus			1.000	1.175	0.833	0.770	0.774	0.369	0.260	0.159
spalax				1.000	0.839	0.737	0.741	0.368	0.231	0.157
xenopus					1.000	0.800	0.808	0.420	0.213	0.132
passer						1.000	1.384	0.504	0.259	0.150
gallus							1.000	0.487	0.272	0.159
timeresurus								1.000	0.413	0.273
stomatitis v									1.000	0.404
parapoxvirus										1.000

MW

	homo	pilocolobus	sus	spalax	xenopus	passer	gallus	timeresurus	stomatitis virus	parapoxvirus
homo	1.000	1.615	1.508	1.363	1.001	0.772	0.785	0.437	0.288	0.153
pilocolobus		1.000	1.511	1.358	0.998	0.772	0.785	0.427	0.290	0.152
sus			1.000	1.366	0.994	0.812	0.825	0.444	0.293	0.162
spalax				1.000	0.950	0.765	0.780	0.404	0.251	0.156
xenopus					1.000	0.807	0.813	0.546	0.252	0.164
passer						1.000	1.475	0.537	0.295	0.116
gallus							1.000	0.519	0.295	0.117
timeresurus								1.000	0.673	0.579
stomatitis v									1.000	0.729
parapoxvirus										1.000

average

	homo	pilocolobus	sus	spalax	xenopus	passer	gallus	timeresurus	stomatitis virus	parapoxvirus
homo	1	1.69	1.61	1.45	1.06	0.90	0.91	0.55	0.37	0.22
pilocolobus		1	1.60	1.44	1.06	0.90	0.90	0.54	0.37	0.22
sus			1	1.46	1.06	0.92	0.93	0.54	0.37	0.22
spalax				1	1.05	0.89	0.90	0.53	0.34	0.22
xenopus					1	0.95	0.97	0.59	0.30	0.21
passer						1	1.61	0.61	0.33	0.17
gallus							1	0.61	0.33	0.18
timeresurus								1	0.62	0.51
stomatitis v									1	0.60
parapoxvirus										1

Figure S7E

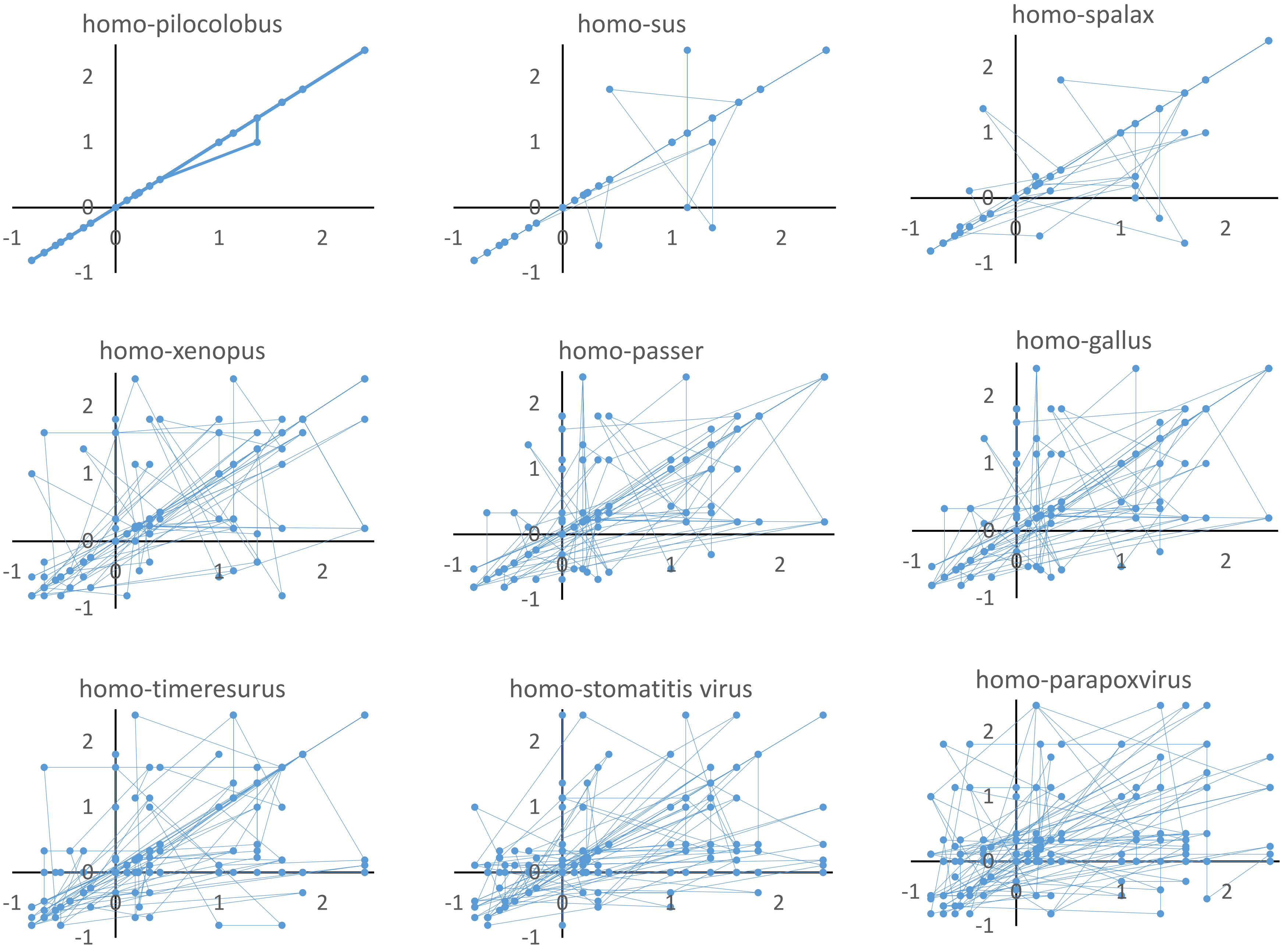
homo-pilocolobus
homo-sus
homo-spalax
homo-xenopus
homo-passer
homo-gallus
homo-timeresurus
homo-stomatitis virus
homo-parapoxvirus

Figure S7F

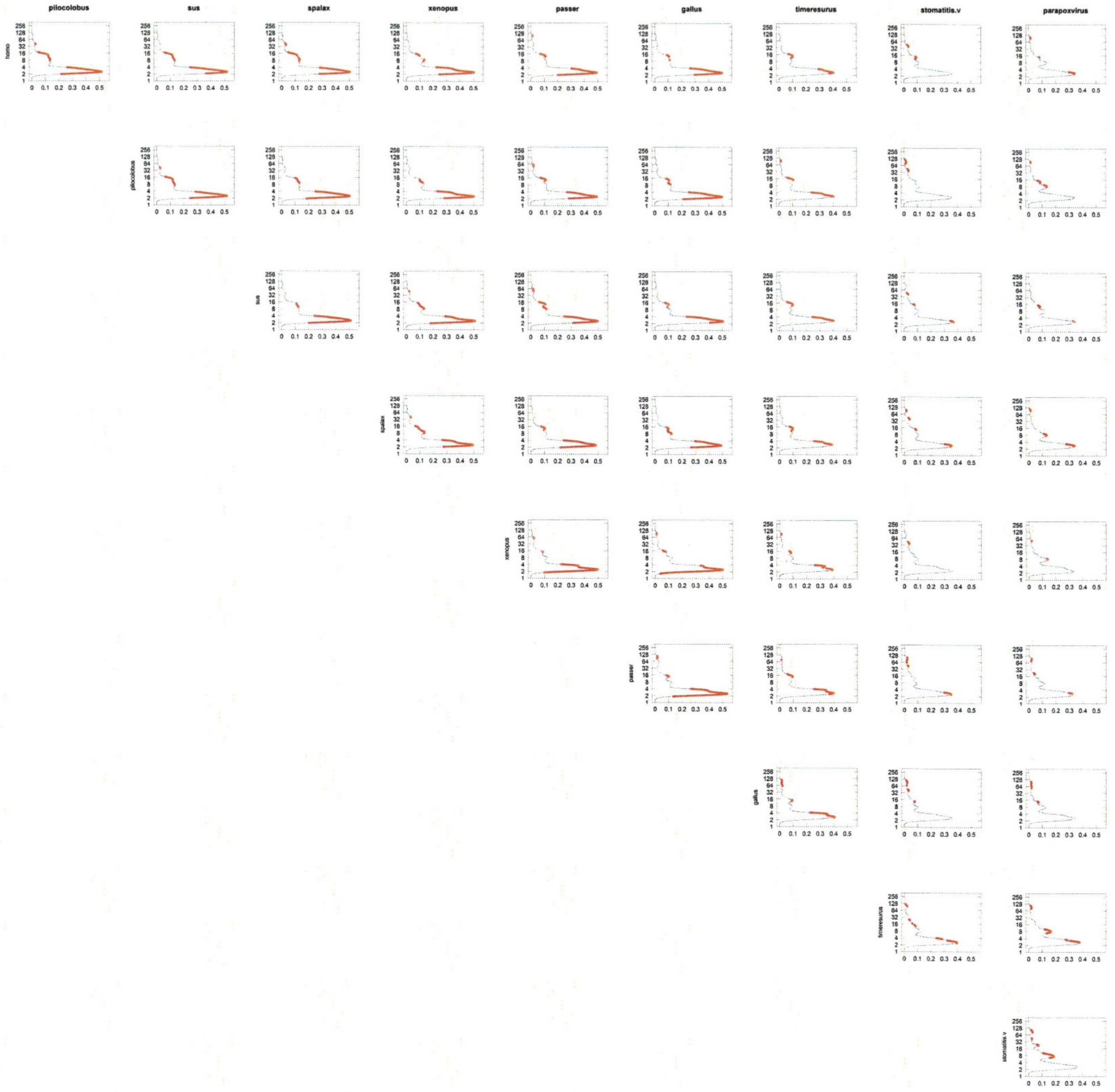
pilocolobus
sus
spalax
xenopus
passer
gallus
timeresurus
stomatitis.v
parapoxvirus
homo
pilocolobus
sus
spalax
xenopus
passer
gallus
timeresurus
stomatitis.v

Figure S7G

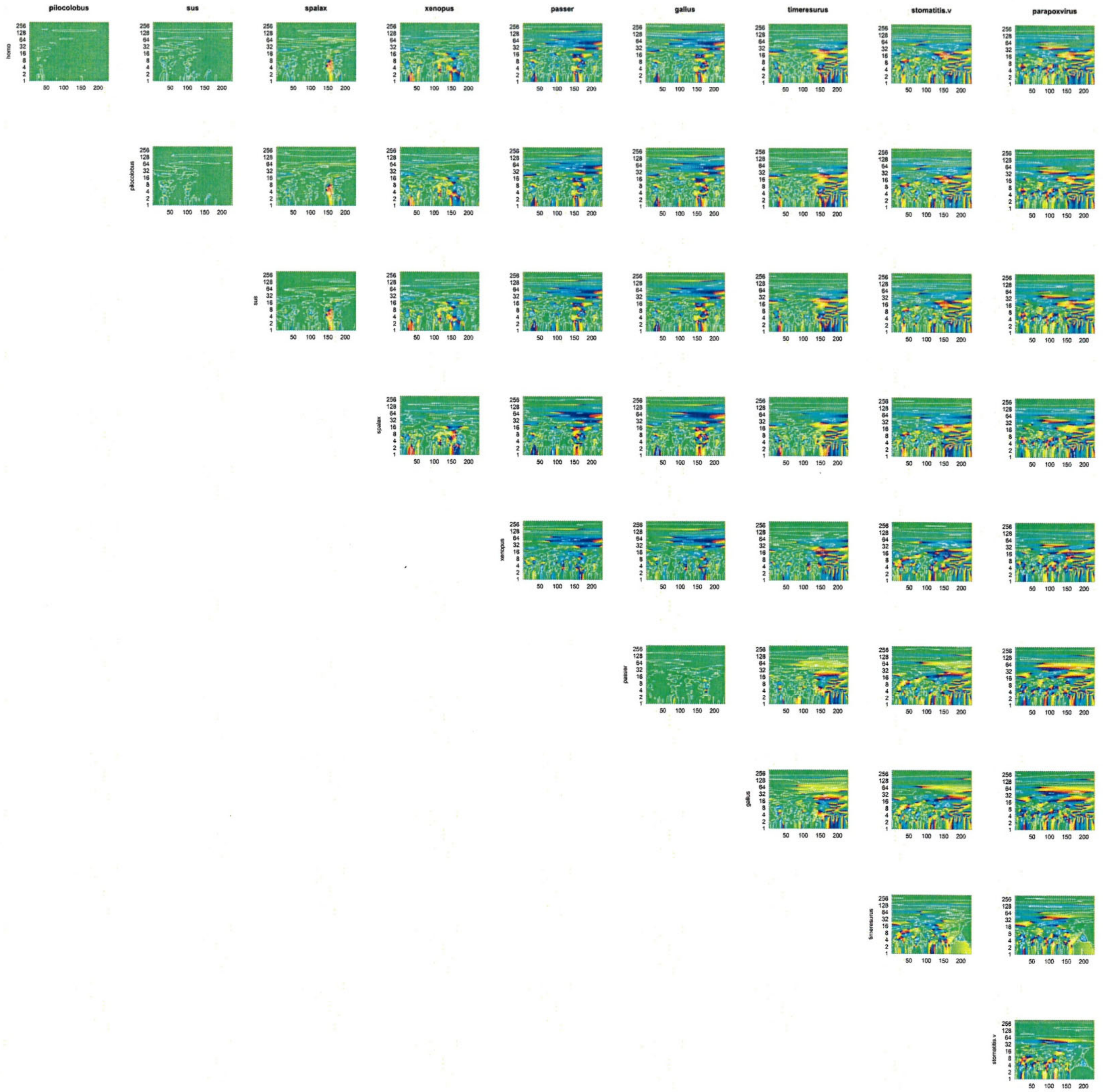

pilocolobus
sus
spalax
xenopus
passer
gallus
timeresurus
stomatitis.v
parapoxvirus
homo
pilocolobus
sus
spalax
xenopus
passer
gallus
timeresurus
stomatitis.v
256
128
64
32
16
8
4
2
1
50
100
150
200

Figure S7H

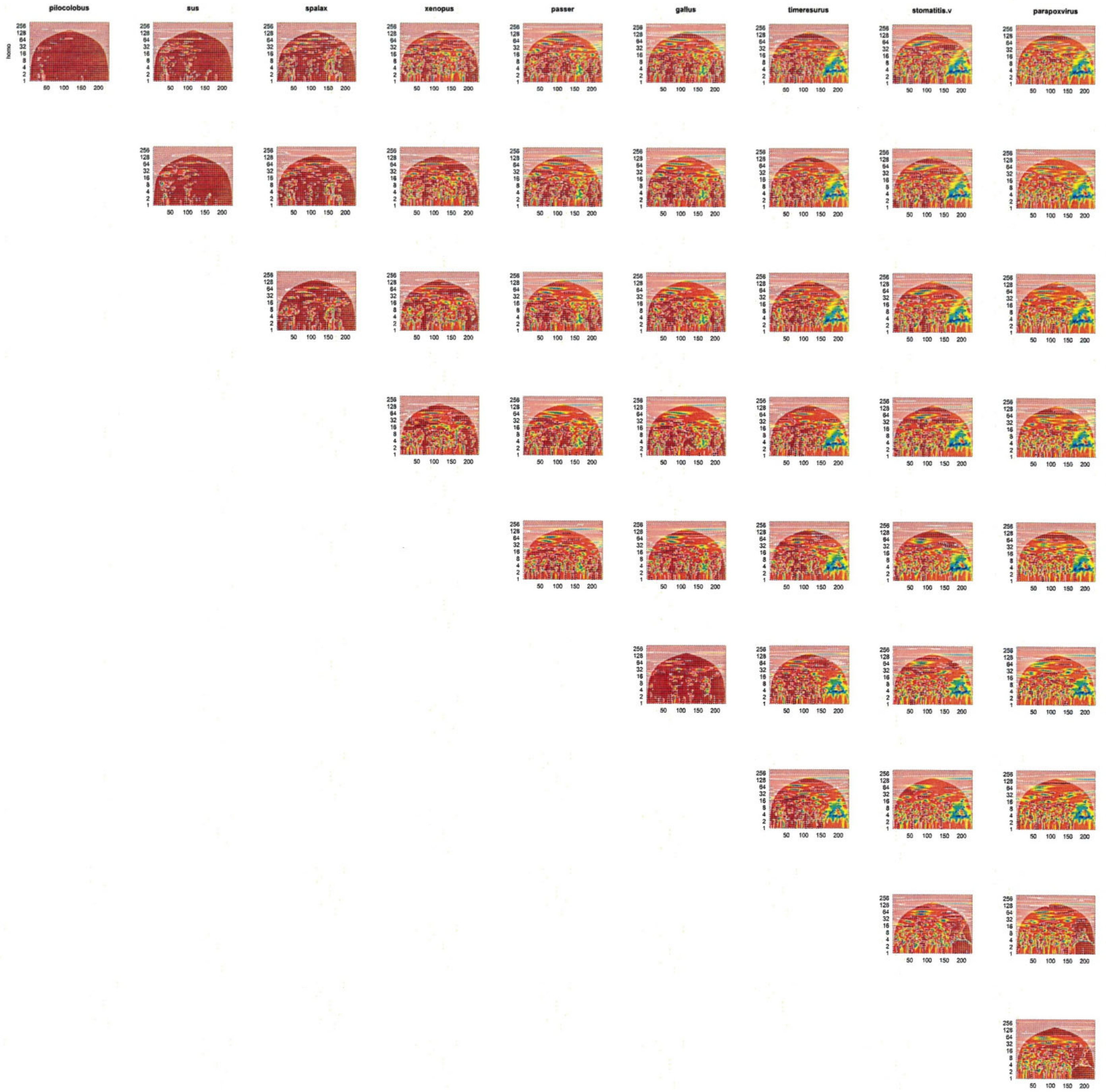
pilocolobus
sus
spalax
xenopus
passer
gallus
timeresurus
stomatitis.v
parapoxvirus
homo

Figure S7I

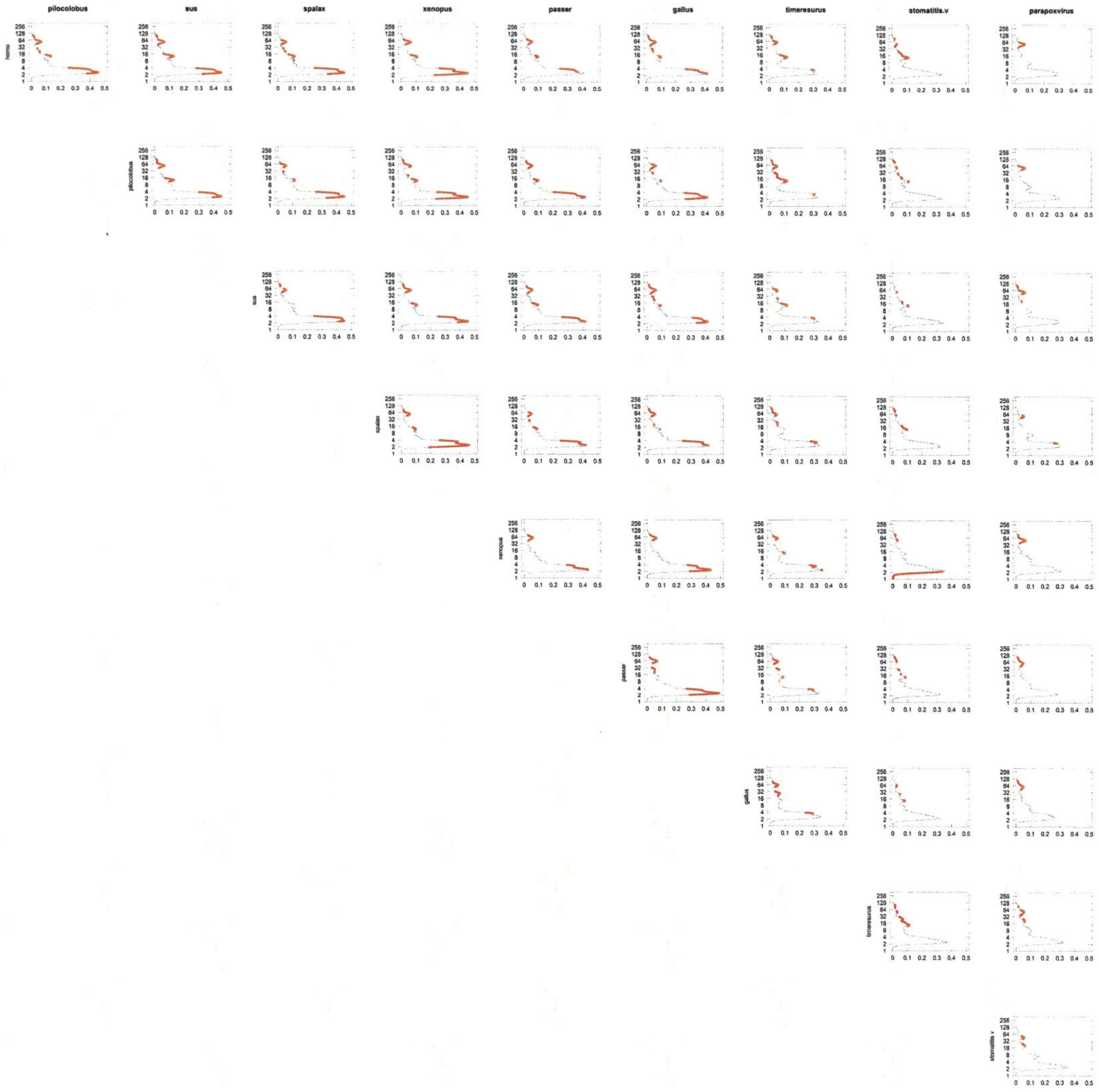

pilocolobus
sus
spalax
xenopus
passer
gallus
timeresurus
stomatitis.v
parapoxvirus
homo
pilocolobus
sus
spalax
xenopus
passer
gallus
timeresurus
stomatitis.v

Figure S7J

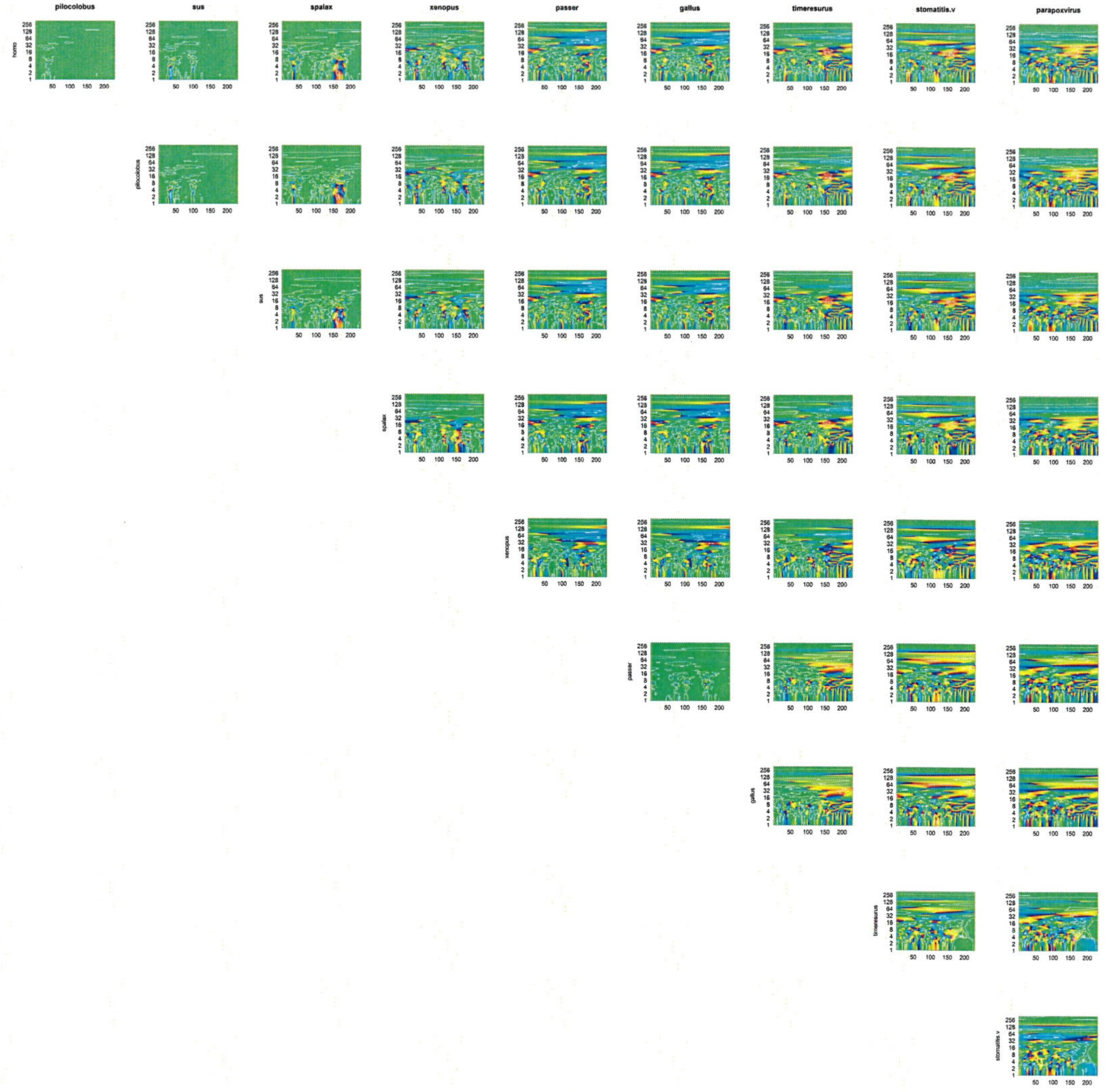

pilocolobus
sus
spalax
xenopus
passer
gallus
timeresurus
stomatitis.v
parapoxvirus
homo
pilocolobus
sus
spalax
xenopus
passer
gallus
timeresurus
stomatitis.v

Figure S7K

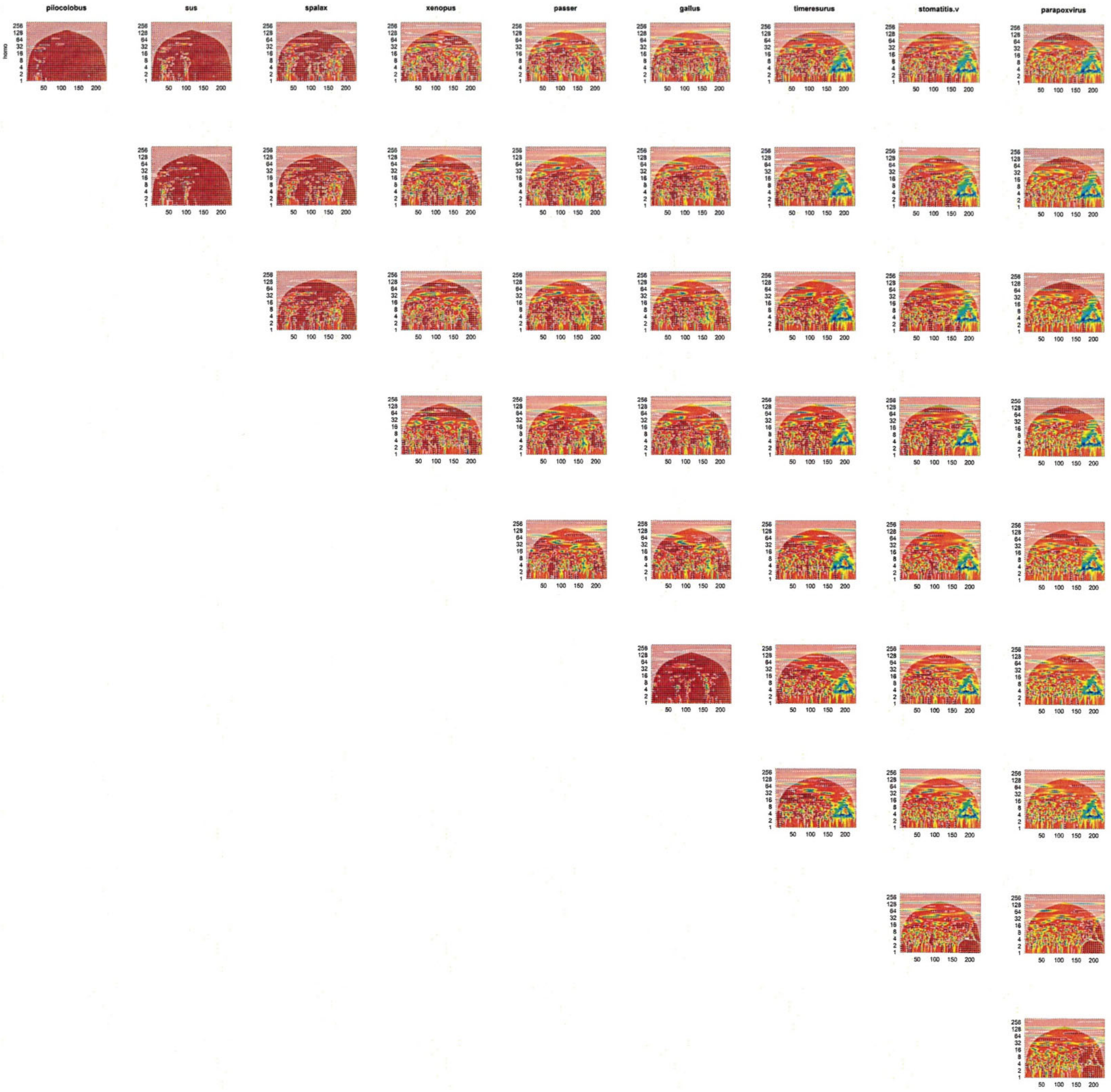
pilocolobus
sus
spalax
xenopus
passer
gallus
timeresurus
stomatitis.v
parapoxvirus
homo

Figure S7L

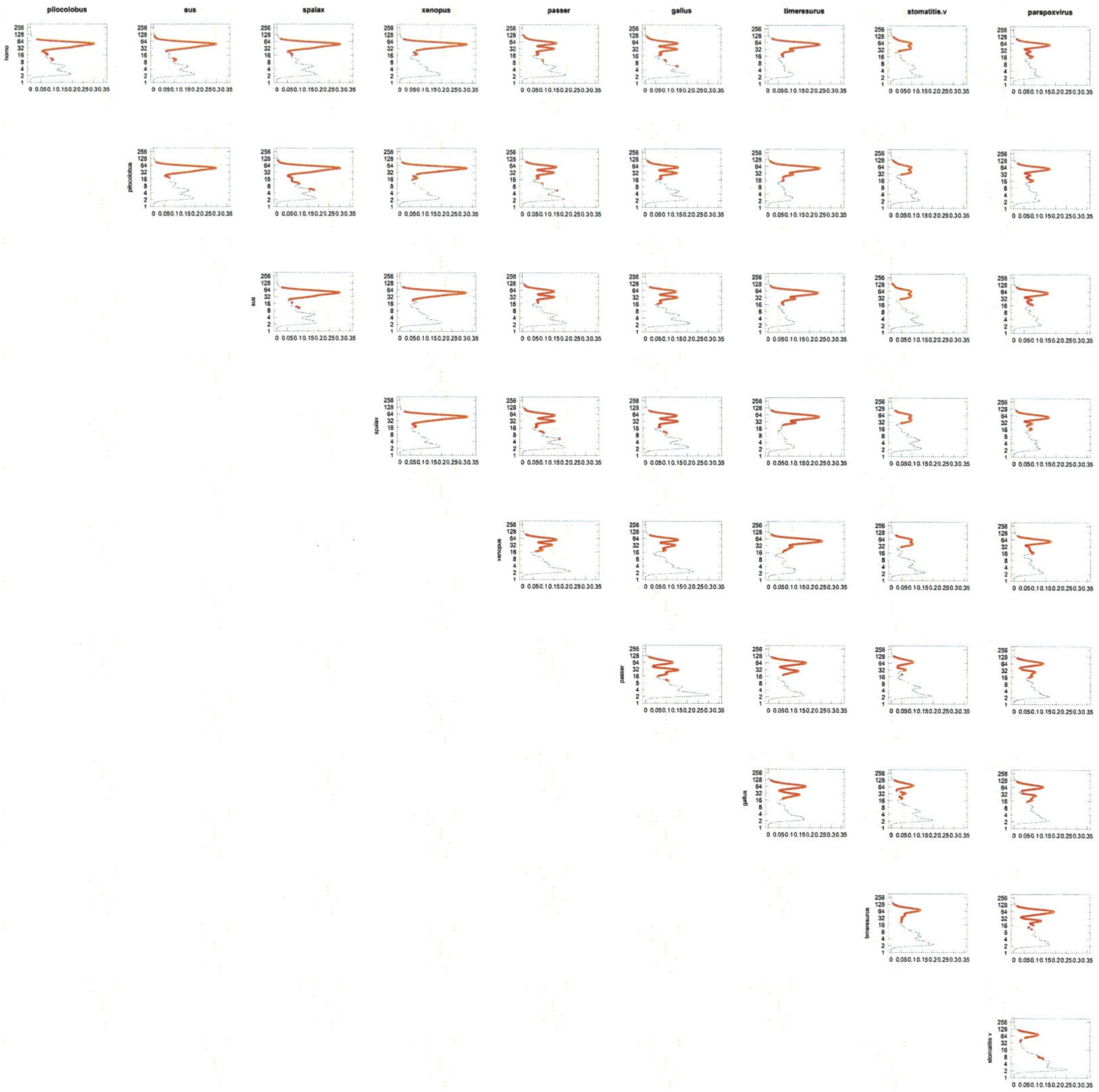
pilocolobus
sus
spalax
xenopus
passer
gallus
timeresurus
stomatitis.v
parapoxvirus
homo
pilocolobus
sus
spalax
xenopus
passer
gallus
timeresurus
stomatitis.v

Figure S7M

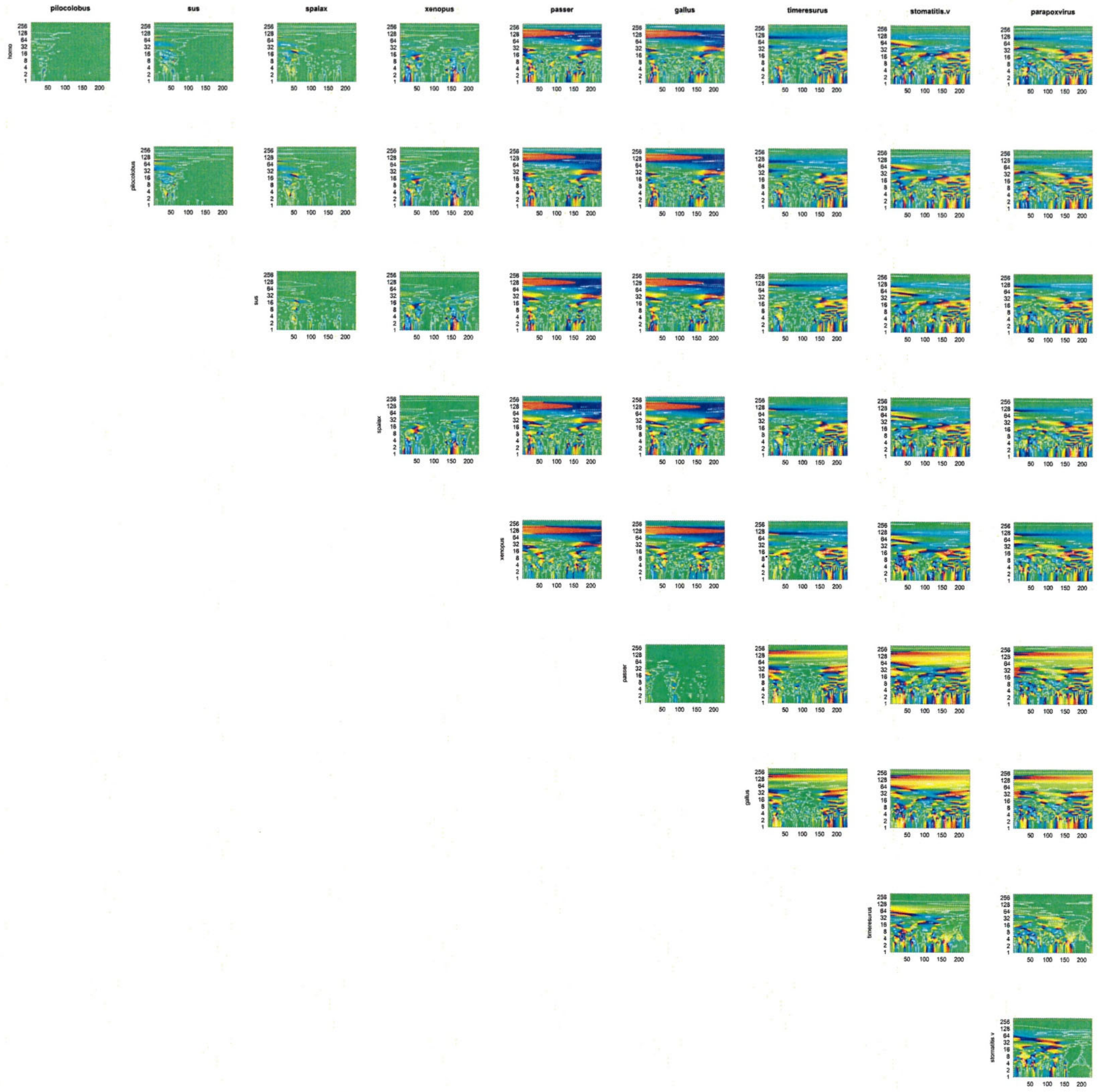

pilocolobus
sus
spalax
xenopus
passer
gallus
timeresurus
stomatitis.v
parapoxvirus
homo
pilocolobus
sus
spalax
xenopus
passer
gallus
timeresurus
stomatitis.v

Figure S7N

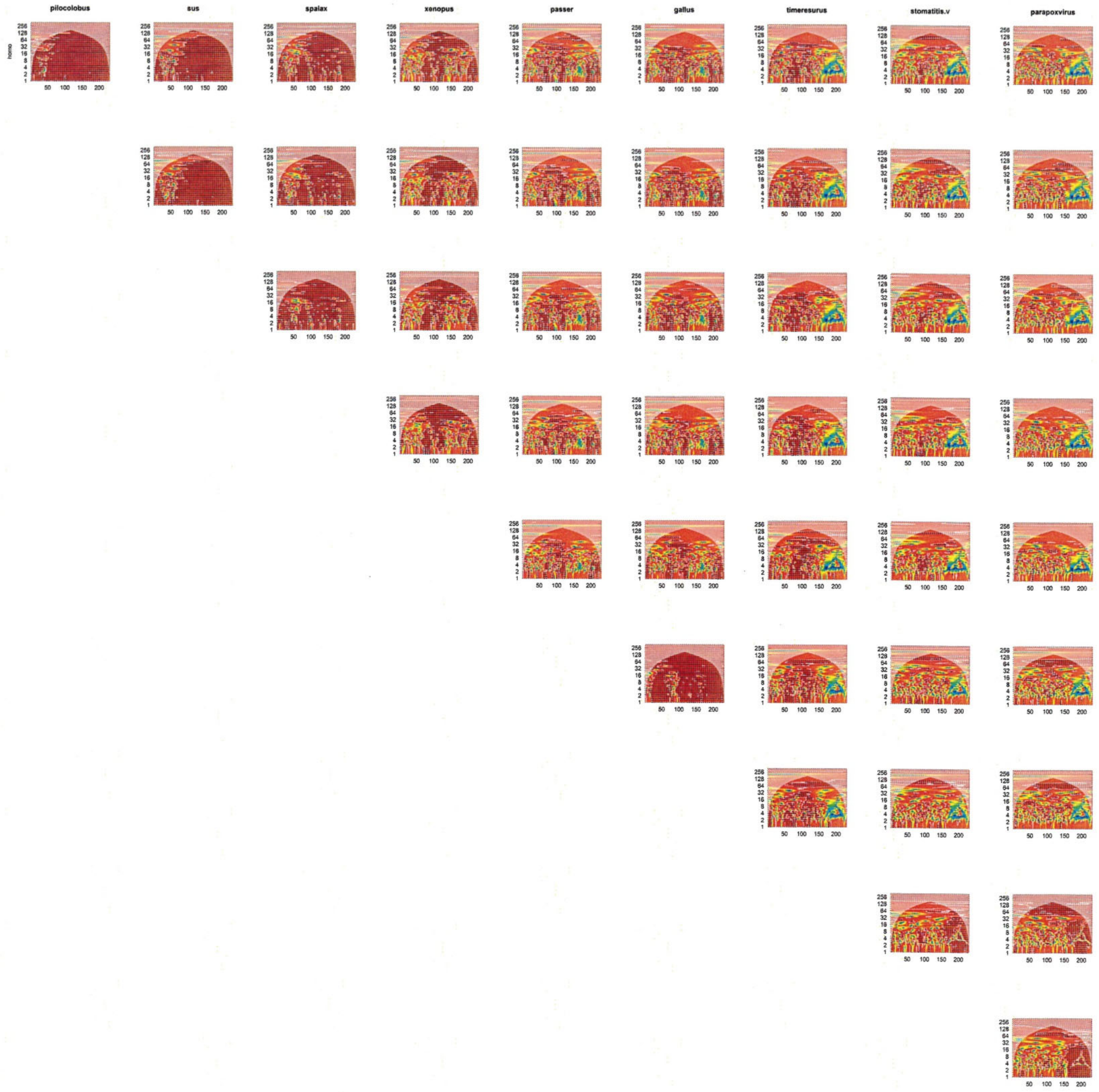
pilocolobus
sus
spalax
xenopus
passer
gallus
timeresurus
stomatitis.v
parapoxvirus
homo
256
128
64
32
16
8
4
2
1
50 100 150 200
